# Role of ammonia for brain abnormal protein glycosylation during the development of hepatitis B virus-related liver diseases

**DOI:** 10.1186/s13578-022-00751-4

**Published:** 2022-02-14

**Authors:** Jiajun Yang, Mengqi Yin, Yao Hou, Hao Li, Yonghong Guo, Hanjie Yu, Kun Zhang, Chen Zhang, Liyuan Jia, Fan Zhang, Xia Li, Huijie Bian, Zheng Li

**Affiliations:** 1 Laboratory for Functional Glycomics, College of Life Sciences, Northwest University, Xi’an, 710069 China; 2 Cell Engineering Research Centre and Department of Cell Biology, Fourth Military Medical University, Xi’an, 710032 China; 3 Department of Infectious Diseases, Second Affiliated Hospital of Xi’an Jiaotong University, Xi’an, 710004 China

**Keywords:** Hepatic encephalopathy, Hyperammonemia, Ca^2+^ homeostasis, C1GALT1, IP3R1

## Abstract

**Background:**

Ammonia is the most typical neurotoxin in hepatic encephalopathy (HE), but the underlying pathophysiology between ammonia and aberrant glycosylation in HE remains unknown.

**Results:**

Here, we used HBV transgenic mice and astrocytes to present a systems-based study of glycosylation changes and corresponding enzymes associated with the key factors of ammonia in HE. We surveyed protein glycosylation changes associated with the brain of HBV transgenic mice by lectin microarrays. Upregulation of Galβ1-3GalNAc mediated by core 1 β1,3-galactosyltransferase (C1GALT1) was identified as a result of ammonia stimulation. Using in vitro assays, we validated that upregulation of C1GALT1 is a driver of deregulates calcium (Ca^2+^) homeostasis by overexpression of inositol 1,4,5-trisphosphate receptor type 1 (IP3R1) in astrocytes.

**Conclusions:**

We demonstrated that silencing C1GALT1 could depress the IP3R1 expression, an effective strategy to inhibit the ammonia-induced upregulation of Ca^2+^ activity, thereby C1GALT1 and IP3R1 may serve as therapeutic targets in hyperammonemia of HE.

**Supplementary Information:**

The online version contains supplementary material available at 10.1186/s13578-022-00751-4.

## Introduction

Hepatic encephalopathy (HE) is a complex neurological syndrome of patients with liver failure, cirrhosis or hepatitis due to severe liver disease or portal-systemic shunts, causing a wide spectrum of neuropsychiatric disorders [1]. HE significantly affects the quality of life and survival of patients, and is also one of the most common causes of death in late-stage liver disease [2]. Pathologically, accumulation of ammonia by liver failure breaks through the blood–brain barrier; glutamine synthesis in astrocytes ensue, causing oxidative stress, changes in osmotic pressure and astrocyte swelling [3]. Although ammonia is the best-characterized neurotoxin in the pathogenesis of HE, the correlative biochemical and cellular changes in the brain are incompletely understood [4].

Glycosylation, the enzymatic process is controlled with the glycosyltransferases and glycosidases by which glycans are attached to proteins and lipids, is the most abundant and functionally important type of post-translational modification [5]. Differential expression of glycosyltransferases in diseases produces abnormal glycosylation related to specific diseases, which interacts with various important biological processes and diseases [6–8]. It is noteworthy that neurons and glia in the central nervous system (CNS) are not only modified with glycans but also depend on their existence and function to perform communication and biological processes, such as brain development, neurodegenerative disorders, psychopathologies, brain cancers, etc. [9]. Currently, there are lack of studies on glycosylation in HE due to difficulties in glycan analysis and complex biosynthetic pathways [10].

Studies of CNS have suggested that neurons transmit electrical and chemical impulses, while glial cells, including astrocytes, microglia, oligodendrocytes and ependymal cells, provide protection, immune defense and support neurons [11]. Astrocytes are the most widely distributed glial cells in the brain, and their dysfunctions are associated with various neurodegenerative disorders [12]. As the regulator of various physiological functions in the brain, the spatiotemporal fluctuation of Ca^2+^ concentration is the major component of astrocytic signaling [13]. The metabotropic glutamate receptor (mGluR) and inositol 1,4,5-trisphosphate receptor (IP3R) long thought to be the main signaling pathway triggering Ca^2+^ events in astrocytes [14, 15]. Of note, the expressions of key components of Ca^2+^ signaling, including metabotropic glutamate receptor 5 (mGluR5) and inositol 1,4,5-trisphosphate receptor type 1 (IP3R1), have been reported to deregulates Ca^2+^ homeostasis in astrocytes [16].

In the present study, we examined the alternations of protein glycosylation in the brain of HBV transgenic mice and in astrocytes stimulated by ammonia and the effects of glycosyltransferase on astrocytic Ca^2+^ homeostasis. We found that the expression levels of galactose type, fucose type, N-acetylglucosamine and mannose type glycans in HBV transgenic mice were remarkably different than those in control mice. Moreover, we showed that C1GALT1, upregulated in NH_4_Cl treated astrocytes associated with Ca^2+^ signaling by targeting IP3R1, demonstrating that aberrant glycosylation can drive neurological dysfunction.

## Results

### Alternations of blood ammonia concentration and astrocytes in HBV transgenic mice

HBV transgenic mice overproduce the hepatitis B virus (HBV) large envelope polypeptide and accumulate toxic quantities of hepatitis B surface antigen (HBsAg) in hepatocyte that develop a severe and persistent hepatocellular injury, leading to precancerous proliferation reaction and unrestrained cell growth [17]. To identify the progress of hepatitis B virus-related liver diseases, alternations of blood ammonia concentration and astrocytes in HBV transgenic mice, we performed HBV transgenic mice C57BL/6J-TG(ALB1HBV)44BRI/J and negative control of C57BL/6 mice.

**Fig. 1:**
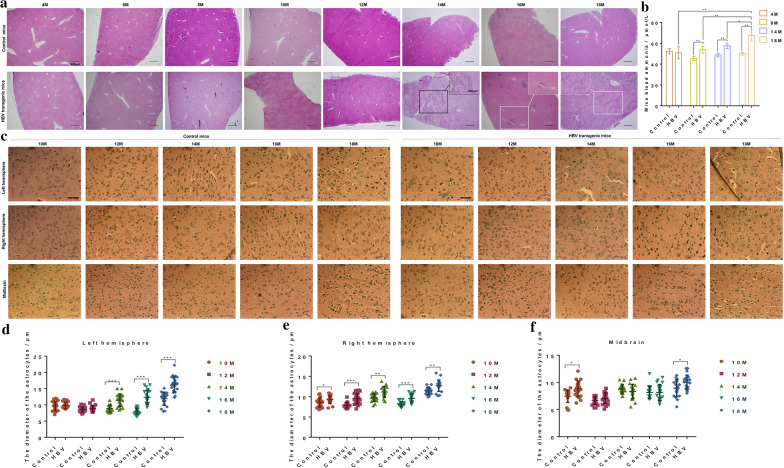
Hepatic injury, blood ammonia concentration and astrocytes in HBV transgenic mice. **a** The H&E staining of mice liver tissues at 4 to 18 months old. Fatty liver was marked with a black box, and liver fibrosis was marked with white a box. Scale bar, 200/500 μm. **b** The serum ammonia concentration of HBV transgenic mice and control mice at 4 to 18 months old. Each group of serum came from a mixture of six mice, at least three replicates per condition. **c** The H&E staining of mice cerebral hemisphere and midbrain at 10 to 18 months old. Scale bar, 50 μm. **d**–**f**, The diameter (1000×) of astrocytes in the left hemispheres (**d**), right hemispheres (**e**) and midbrain (**f**)

The H&E staining of mice liver tissues showed that the livers of control mice aged 4 to 18 months were normal, while hepatocellular overexpression of HBV protein leaded to severe and persistent hepatocellular injury in HBV transgenic mice (Fig. 1a). With the HBV accumulation, the liver of HBV transgenic mice aged 4 to14 months gradually progressed fatty liver; Local liver fibrosis in 16 months old HBV transgenic mice; The liver of 18 months old HBV transgenic mice developed extensive liver fibrosis and cirrhosis (Fig. 1a).

Ammonia is a neurotoxin related to the pathogenesis of HE and plays an important role in astrocyte swelling, so we compared the serum ammonia of HBV transgenic mice and control mice. The serum ammonia concentration of HBV transgenic mice from 4 to 18 months old was significantly increased and was significantly higher than control mice at 8 to 18 months old (Fig. 1b).

In order to observe the distribution of cells in the brain of mice, the H&E staining of mice heads tissues showed that granulosa cells were mainly found in the olfactory bulb and cerebellum (Additional file 1: Fig. S1), and astrocytes were mainly observed in cerebral hemisphere and midbrain (Fig. 1c). The intracellular accumulation of glutamine leads to astrocyte swelling, eventually leads to neurological dysfunction [18]. The results in Fig. 1c only indicated that the distribution of cells in HBV transgenic mice and control mice was consistent, but whether the astrocyte swelling could not be determined directly from the images. To further examine the swelling of astrocytes, we measured the diameter of astrocytes at 1000 times magnification. The diameter of astrocytes in the left and right hemispheres of HBV transgenic mice aged 14/10 to 18 months was significantly larger than the control mice (Fig. 1d–e), and the diameter of astrocytes in the midbrain of HBV transgenic mice aged 10 and 18 months was significantly larger than the control mice (Fig. 1f). These results demonstrated that the serum ammonia concentration was up-regulated, and astrocytes were swollen during the development of hepatic disease in HBV transgenic mice.

### Alterations of tissue glycopatterns in the brain of HBV transgenic mice

Although abnormal glycosylation has been reported in various diseases, these studies have focused on tumor to exclude brain complications of chronic hepatic disease. Therefore, we sought to determine the alternations of glycosylation in the brain of HBV transgenic mice. The protein of olfactory bulb, left hemisphere, right hemisphere, midbrain and cerebellum from HBV transgenic mice (n = 6, at the same age) and control mice (n = 6, at the same age) were measured by lectin microarrays, which included 37 lectin probes, providing specific information for the glycan repertoire of tissue glycoproteins [19]. The layout of the lectin microarrays is shown in Additional file 1: Fig. S2.

The glycopatterns of proteins in olfactory bulb from HBV transgenic mice and control mice bound to the lectin microarrays and their normalized fluorescent intensities (NFIs) for each lectin are shown in Additional file 1: Fig. S3a. The generated data from each mouse was imported into EXPANDER 6.0 to perform a hierarchical clustering analysis (Additional file 1: Fig. S3b). The results showed the (GlcNAc)_2–4_ binder LEL, the α-D-Man, Fucα-1,6GlcNAc, α-D-Glc binder PSA, Fucα-1,6GlcNAc, α-D-Man, α-D-Glc binder LCA and the High-Mannose, Manα1-6Man binder NPA, exhibited significantly altered NFIs in the olfactory bulb of HBV transgenic mice compared with control mice (Additional file 1: Fig. S3c).

**Fig. 2:**
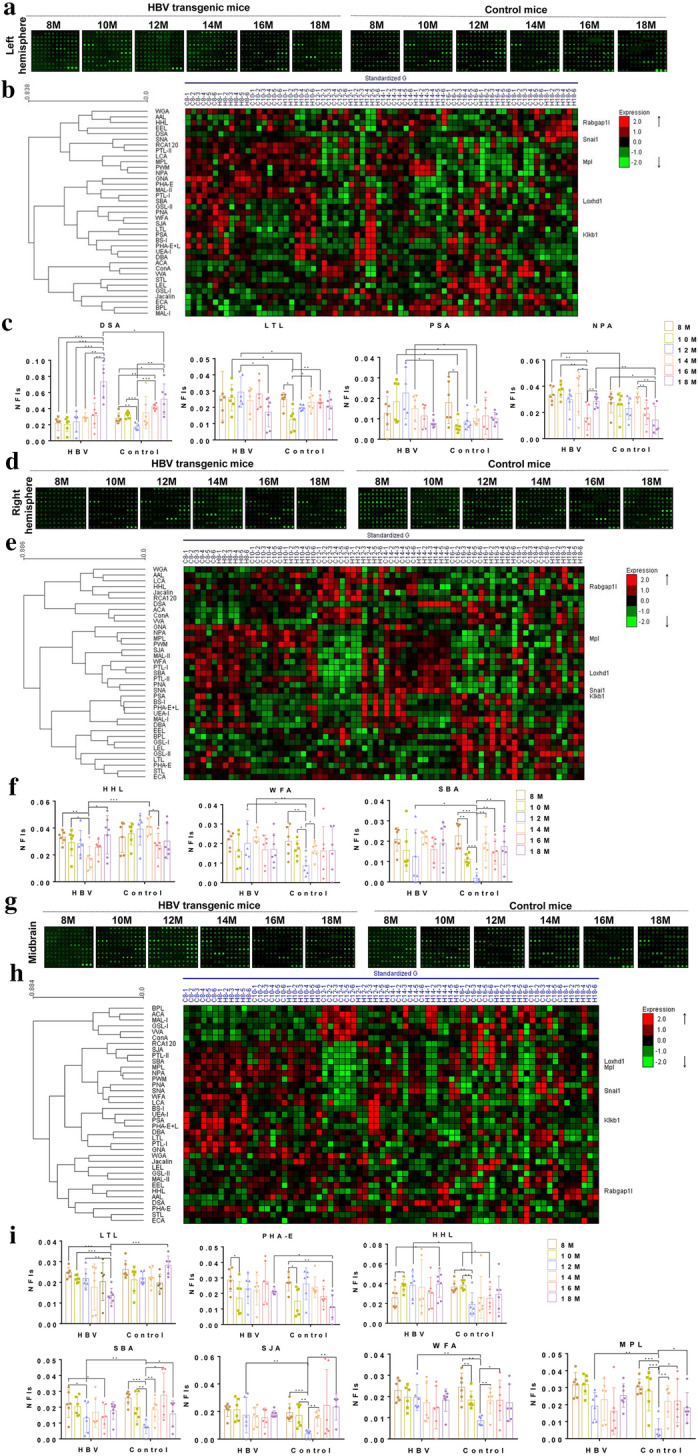
The different tissue glycopatterns in HBV transgenic mice and control mice using lectin microarrays. **a** The glycopatterns of Cy3-labeled left hemisphere samples bound to the lectin microarrays. **b** Heat map and hierarchical clustering of the 37 lectins in glycopatterns of left hemisphere. **c** Four lectins revealed significant differences glycopatterns in left hemisphere between HBV transgenic mice and control mice. **d** The glycopatterns of Cy3-labeled right hemisphere samples bound to the lectin microarrays. **e** Heat map and hierarchical clustering of the 37 lectins in glycopatterns of right hemisphere. **f** Three lectins revealed significant differences glycopatterns in right hemisphere between HBV transgenic mice and control mice. **g** The glycopatterns of Cy3-labeled midbrain samples bound to the lectin microarrays. **h** Heat map and hierarchical clustering of the 37 lectins in glycopatterns of midbrain. **i** Seven lectins revealed significant differences glycopatterns in midbrain between HBV transgenic mice and control mice. In the heat map and hierarchical clustering, the samples were listed in columns and the lectins were listed in rows, and the color of each square represented the expression levels relative to the other data (Red, high; green, low; black, medium). Data shown are representative of six independent replicates

The glycopatterns of proteins in left hemisphere from HBV transgenic mice and control mice bound to the lectin microarrays, as shown in Fig. 2a. Their NFIs for each lectin are showed in Additional file 1: Fig. S4a. The generated data from each mouse were imported into EXPANDER 6.0 to perform a hierarchical clustering analysis (Fig. 2b). The results showed the β-D-GlcNA, (GlcNAcβ1-4)_n_, Galβ1-4GlcNAc binder DSA, the α-D-Man, the High-Mannose, Manα1-6Man binder NPA, the Fucα1-2Galβ1-4GlcNAc, Fucα1-3(Galβ1-4)GlcNAc, anti-H blood group specificity binder LTL and the Fucα-1,6GlcNAc, α-D-Glc binder PSA, exhibited significantly altered NFIs in left hemisphere of HBV transgenic mice compared with control mice (Fig. 2c).

The glycopatterns of proteins in right hemisphere from HBV transgenic mice and control mice bound to the lectin microarrays, as shown in Fig. 2d. Their NFIs for each lectin are summarized in Additional file 1: Fig. S4b. The generated data from each mouse was imported into EXPANDER 6.0 to perform a hierarchical clustering analysis (Fig. 2e). The results showed the High-Mannose, Manα1-3Man, Manα1-6Man, Man5-GlcNAc2-Asn binder HHL, the α-/β-linked terminal GalNAc, (GalNAc)_n_, GalNAcα1-3Gal, blood-group A binder SBA and the terminating in GalNAcα/β1-3/6Gal binder WFA, exhibited significantly altered NFIs in right hemisphere of HBV transgenic mice compared with control mice (Fig. 2f).

The glycopatterns of proteins in midbrain from HBV transgenic mice and control mice bound to the lectin microarrays, as shown in Fig. 2g. Their NFIs for each lectin are summarized in Additional file 1: Fig. S4c. The generated data from each mouse was imported into EXPANDER 6.0 to perform a hierarchical clustering analysis (Fig. 2h). The results showed the Fucα1-2Galβ1-4GlcNAc, Fucα1-3(Galβ1-4)GlcNAc, anti-H blood group specificity binder LTL, the Bisecting GlcNAc, biantennary complex-type N-glycan binder PHA-E, the High-Mannose, Manα1-3Man, Manα1-6Man, Man5-GlcNAc2-Asn binder HHL, the α-/β-linked terminal GalNAc, (GalNAc)_n_, GalNAcα1-3Gal, blood-group A binder SBA, the Terminal in GalNAc and Gal, anti-A and anti-B human blood group binder SJA, the terminating in GalNAcα/β1-3/6Gal binder WFA and the Galβ1-3GalNAc binder MPL, exhibited significantly altered NFIs in the midbrain of HBV transgenic mice compared with control mice (Fig. 2i).

The glycopatterns of proteins in cerebellum from HBV transgenic mice and control mice bound to the lectin microarrays and their NFIs for each lectin are shown in Additional file 1: Fig. S3d. The generated data from each mouse were imported into EXPANDER 6.0 to perform a hierarchical clustering analysis (Additional file 1: Fig. S3e). The results showed that the NFIs did not exhibit significantly in the midbrain of HBV transgenic mice compared with control mice.

Next, protein microarrays and lectin blotting analyses were performed using LEL selected randomly to confirm the different abundances of glycopatterns in pooled tissues from olfactory bulb. The results of protein microarrays showed that the glycopatterns recognized by LEL from HBV transgenic mice exhibited higher expression than control mice (Additional file 1: Fig. S5a–b). The results of SDS-PAGE demonstrated that the protein bands from HBV transgenic mice and control mice were similar (Additional file 1: Fig. S5c). The intensity of LEL bond to protein of olfactory bulb was consistent with NFIs trend of lectin microarrays in HBV transgenic mice and control mice (Additional file 1: Fig. S5d–e).

To further validate and assess the distribution and localization of specific glycans, including galactose type, fucose type (Fuc), N-acetylglucosamine (GlcNAc) and mannose type glycans in HBV transgenic mice and control mice, the fluorescence-based lectin histochemistry was performed using three lectins selected randomly (NPA, PSA and SBA), according to our previous protocol [20]. The negative controls showed no positive signal (Additional file 1: Fig. S6a), and the selected lectins showed various binding patterns (Additional file 1: Fig. S6b–g). The High-Mannose, Manα1-6Man recognized by NPA showed strong binding to the nuclear and cytoplasmic regions of granulosa cells in olfactory bulb (Additional file 1: Fig. S6b), while NPA mainly bond to the cytoplasmic regions in the left hemisphere (Additional file 1: Fig. S6c). The α-D-Man, Fucα-1,6GlcNAc, α-D-Glc recognized by PSA showed strong binding to the cytoplasmic and membrane areas in the olfactory bulb (Additional file 1: Fig. S6d) and left hemisphere (Additional file 1: Fig. S6e). Galactose type recognized by SBA showed strong binding to the cell membrane, and little binding to cytoplasmic regions in the left hemisphere (Additional file 1: Fig. S6f) and midbrain (Additional file 1: Fig. S6g).

These results demonstrated that the protein glycopatterns identified by 12 lectins (MPL, PSA, SJA, LCA, PHA-E, DSA, LEL, SBA, LTL, NPA, HHL and WFA) exhibited significantly changed NFIs in the brain of HBV transgenic mice compared with control mice. Notably, a total of 10 lectins (MPL, PSA, SJA, PHA-E, DSA, SBA, LTL, NPA, HHL and WFA) showed significant differences in the cerebral hemispheres and midbrain, where astrocytes were widely expressed. The sugar-binding specificities of the 10 altered lectins in HBV transgenic mice are shown in Additional file 1: Table S1.

### Effects of NH_4_Cl on protein glycosylation in astrocytes

**Fig. 3:**
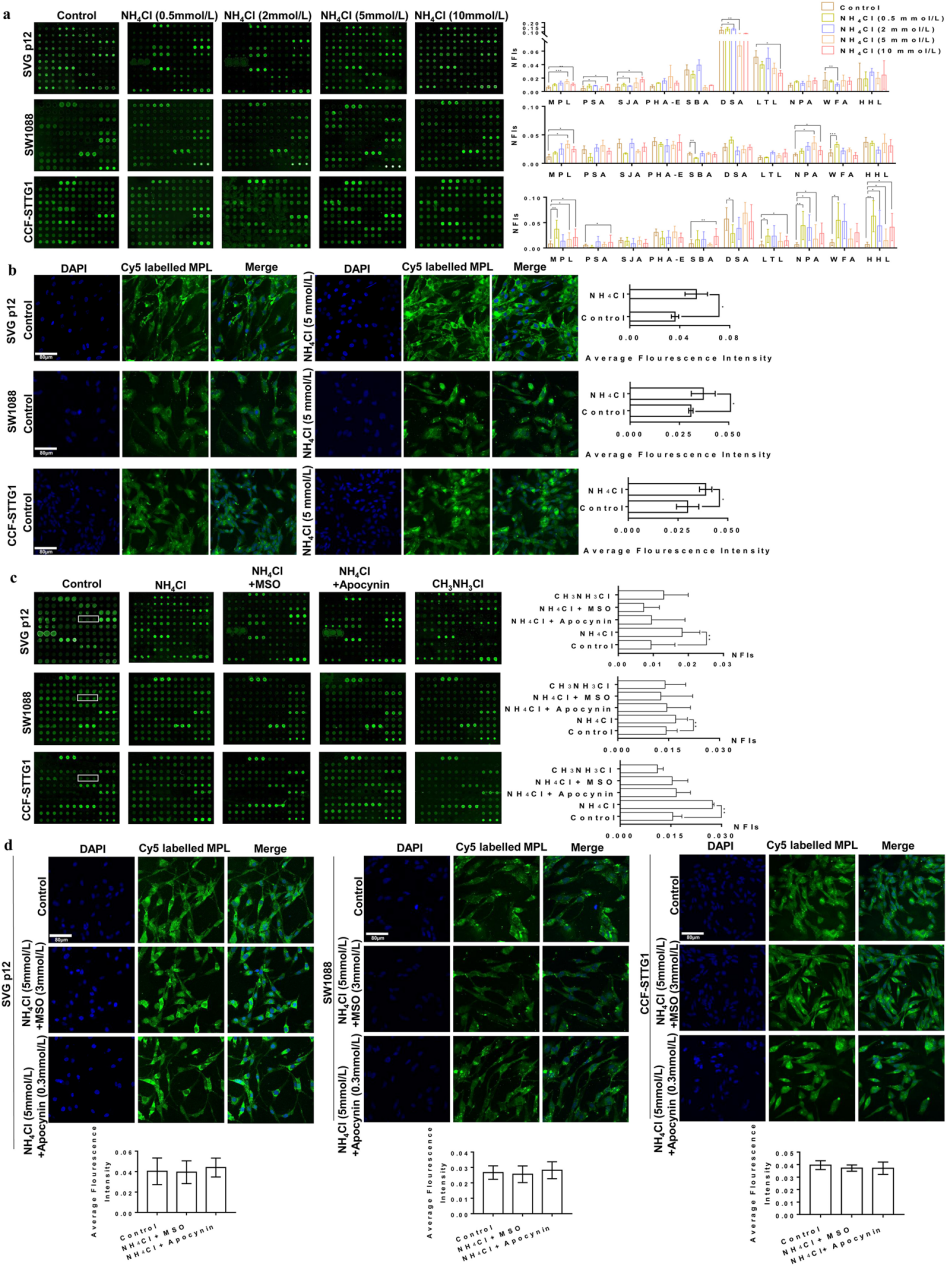
Ammonia-induced alternations of protein glycosylation in astrocytes. **a** The glycopatterns of Cy3-labeled protein of SVG p12, SW1088 and CCF-STTG1 cells bound to the lectin microarrays, and the NFIs of 10 lectins in the 0.5, 2, 5 or 10 mmol/L NH_4_Cl treated and untreated cells. **b** The fluorescence-based lectin cytochemical and average fluorescence intensity of Galβ1-3GalNAc binder MPL in 5 mmol/L NH_4_Cl treated compared with untreated SVG p12, SW1088 and CCF-STTG1 cells. **c** The glycopatterns of Cy3-labeled protein of SVG p12, SW1088 and CCF-STTG1 cells bound to the lectin microarrays and their NFIs, cells were treated with NH_4_Cl plus the glutamine synthetase inhibitor (L-Methionine sulfoximine, MSO), NADPH oxidase inhibitor (apocynin) or CH_3_NH_3_Cl. MPL was marked in the white boxes. **d** The fluorescence-based lectin cytochemical and average fluorescence intensity of Galβ1-3GalNAc binder MPL in SVG p12, SW1088 and CCF-STTG1 cells, which treated with NH_4_Cl plus the glutamine synthetase inhibitor (L-Methionine sulfoximine, MSO) or NADPH oxidase inhibitor (apocynin). The images were acquired using the same condition and shown on the same scale in the Cy5- and DAPI-merge channel, Scale Bar 80 μm

Ammonia is the most typical neurotoxin in the pathogenesis of HE, which could be synthesized into glutamine in astrocytes [21]. However, the relevance between ammonia and glycosylation changes in astrocytes remains unknown. We hypothesized that the abnormal glycosylation patterns observed in the brain of HBV transgenic mice could contribute to the ammonia concentration. To test this hypothesis, we utilized lectin microarrays to measure the effect of NH_4_Cl on protein glycosylation in astrocytes (SVG p12, SW1088 and CCF-STTG1). After serum-free for 24 h, astrocytes were left untreated for 72 h or treated with 0.5, 2, 5 or 10 mmol/L NH_4_Cl for 72 h. The medium was changed daily to ensure the concentration of NH_4_Cl. The NFIs for 37 lectins in astrocytes are summarized in Additional file 1: Fig. S7. The NFIs of 10 lectins, which altered in the brain of HBV transgenic mice, in the NH_4_Cl treated astrocytes and untreated astrocytes are shown in Fig. 3a. The preferred specificity of Maclura pomifera lectin (MPL) is Galβ1-3GalNAc structure, so MPL is called binder of Galβ1-3GalNAc structure. The binding amount of MPL with Galβ1-3GalNAc structure could reflected by the fluorescence intensity to indicate its expression level [19]. The results showed that the Galβ1-3GalNAc binder MPL exhibited significantly increased NFIs in NH_4_Cl treated compared with untreated SVG p12, SW1088 and CCF-STTG1 cells (Fig. 3a), which was consistent with the changing trend in the brain of HBV transgenic mice. This result suggested that ammonia affected the expression of Galβ1-3GalNAc.

Fluorescence-based lectin cytochemistry is an experimental technique similar to lectin microarrays, which could also reflect the binding amount of MPL with Galβ1-3GalNAc structure via the fluorescence intensity to indicate its expression level. Next, we validated the distribution of Galβ1-3GalNAc in NH_4_Cl treated astrocytes by fluorescence-based lectin cytochemistry. It was suggested that astrocytes were treated with 5 mmol/L NH_4_Cl, a concentration of ammonia was found in the brains of experimental hepatic failure [22]. The negative controls showed no positive signal (Additional file 1: Fig. S8a). Consistent with our observations in lectin microarrays, the expression levels of Galβ1-3GalNAc recognized by MPL increased significantly in 5 mmol/L NH_4_Cl treated in comparison with untreated SVG p12, SW1088 and CCF-STTG1 cells (Fig. 3b).

Consequently, to test for the reversibility of NH_4_Cl-induced Galβ1-3GalNAc, SVG p12 cells were left untreated or exposed to NH_4_Cl (5 mmol/L) for 72 h followed by another incubation for 72 h in an NH_4_Cl-free culture medium. The results showed that Galβ1-3GalNAc returned to basal level when NH_4_Cl containing medium was removed after 72 h (Additional file 1: Fig. S8b–c). Our finding provided evidence that the increase of Galβ1,3-GalNA related to the upgrade of ammonia concentration.

### Effects of glutamine, nicotinamide adenine dinucleotide phosphate (NADPH) and pH changes on Galβ1-3GalNAc in astrocytes

Ammonia accumulated in the blood can cross the blood–brain barrier into astrocytes, metabolized into glutamine by glutamine synthetase [23]. Ammonia-induced glutamine accumulation in astrocytes is a HE marker in patients with liver cirrhosis. To test the role of NH_4_Cl (5 mmol/L, 72 h) induced glutamine formation on the expression of Galβ1-3GalNAc, astrocytes were treated with NH_4_Cl and the glutamine synthetase inhibitor (L-Methionine sulfoximine, MSO, 3 mmol/L). In liver cirrhosis, HE is accompanied by oxidative stress due to the activation of NADPH oxidase [24]. The synergistic relationship between oxidative stress and hyperammonemia is essential for brain edema in HE [25]. To test for the role of NH_4_Cl (5 mmol/L, 72 h) induced NADPH on Galβ1-3GalNAc, astrocytes were exposed to NH_4_Cl and NADPH oxidase inhibitor (apocynin, 0.3 mmol/L). To determine whether NH_4_Cl-induced intracellular pH changes trigger increased Galβ1-3GalNAc, astrocytes were treated with CH_3_NH_3_Cl (5 mmol/L) for 72 h, which is a compound that produces the same intracellular pH changes as NH_4_Cl [26, 27]. As shown in Fig. 3c, inhibition of glutamine synthetase and NADPH oxidase significantly prevented the NH_4_Cl mediated upgrade of Galβ1-3GalNAc, and exposure to CH_3_NH_3_Cl did not affect the expression of Galβ1-3GalNAc.

To further validate and assess the expression of Galβ1-3GalNAc, fluorescence-based lectin cytochemistry was performed. Consistent with our observations in lectin microarrays, inhibition of glutamine synthetase and NADPH oxidase completely blocked the upgrade levels of Galβ1-3GalNAc in NH_4_Cl treated SVG p12, SW1088 and CCF-STTG1 cells (Fig. 3b and d), while astrocytes were exposed to CH_3_NH_3_Cl did not affect Galβ1-3GalNAc (Additional file 1: Fig. S9). These findings suggest that ammonia up-regulated Galβ1-3GalNAc in astrocytes in a glutamine synthesis- and NADPH oxidase-dependent manner but was independent of ammonia-induced intracellular pH changes.

### Ammonia stimulated C1GALT1 expression and intracellular calcium activity in astrocytes

**Fig. 4:**
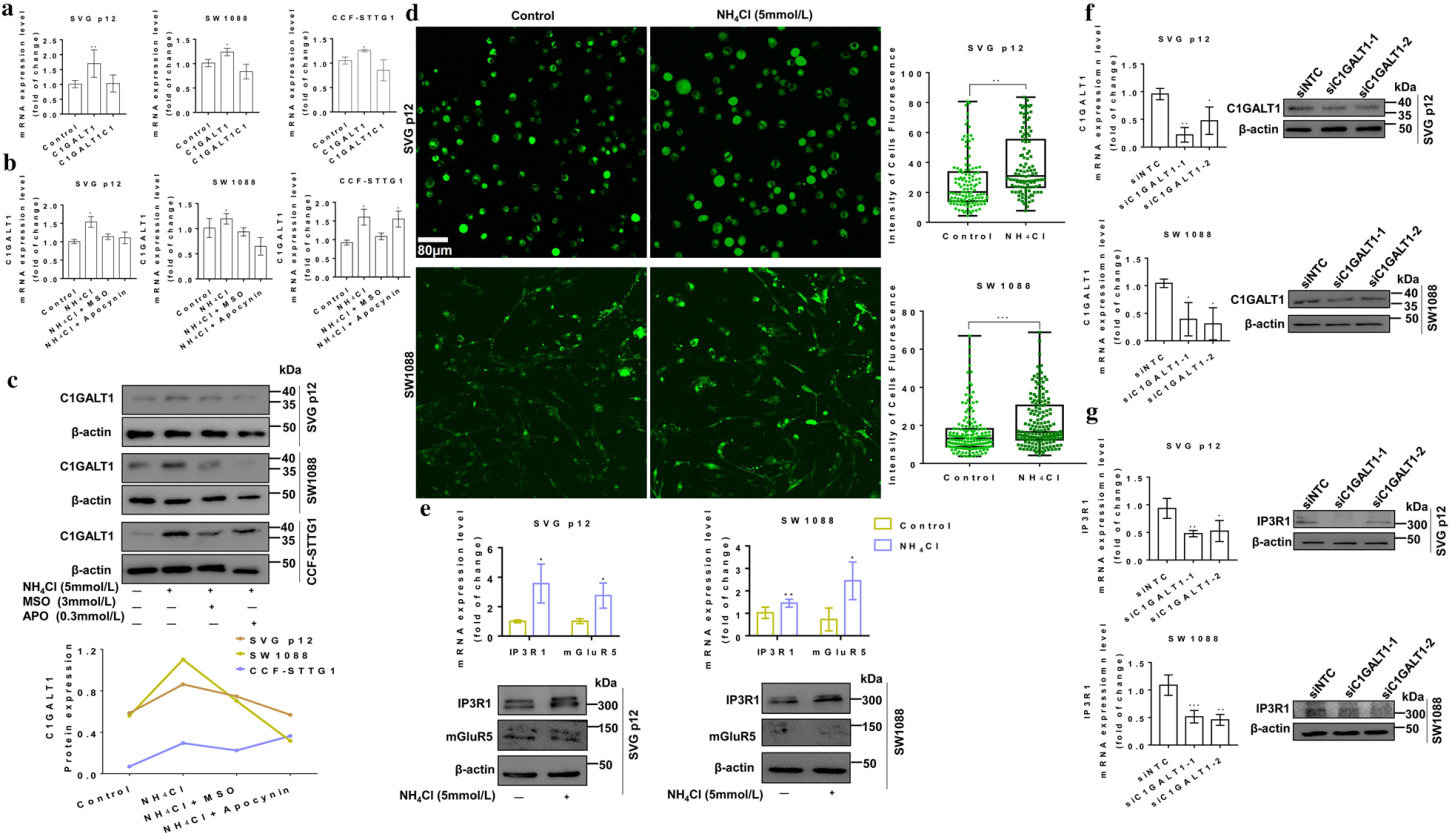
Ammonia stimulated C1GALTI expression and C1GALT1 silencing in astrocytes. **a** CIGALT1 and C1GALT1C1 mRNA levels in NH_4_Cl treated SVG p12, SW1088 and CCF-STTG1 cells were assessed by real-time PCR. **b** CIGALT1 mRNA levels in NH_4_Cl plus MSO or apocynin treated SVG p12, SW1088 and CCF-STTG1 cells were assessed by real-time PCR. **c** Western blotting of C1GALT1 protein levels in NH_4_Cl plus MSO or apocynin treated astrocytes compared with untreated astrocytes. **d** Ca^2+^ was loaded with Fluo-4 AM in NH_4_Cl treated and untreated SVG p12 and SW1088 cells and their fluorescence intensity of Ca^2+^. Fluorescent images were captured using the same condition, Scale Bar 80 μm. **e** Real-time PCR and western blotting of IP3R1 and mGluR5 levels in NH_4_Cl treated and untreated SVG p12 and SW1088 cells. **f** C1GALT1 levels in SVG p12 and SW1088 cells expressing siRNA targeting C1GALT1 (siC1GALT1-1 or siC1GALT1-2) or non-targeting control (siNTC) were determined by real-time PCR and western blotting. **g** IP3R1 levels of SVG p12 and SW1088 cells transduced with siC1GALT1-1, siC1GALT1-2 or siNTC were assessed by real-time PCR and western blotting. Real-time PCR graph shows average relative expression normalized to GAPDH, and data are from at three independent cultures performed in least three times

Glycosyltransferases have known to be involved in the synthesis of glycan epitopes. Glycosyltransferases of C1GALT1 and it’ s molecular chaperone (C1GALT1C1) are responsible for the synthesis of Galβ1-3GalNAc [28]. To gain insight into the biosynthetic underpinnings of the up-regulated Galβ1-3GalNAc, the expression of C1GALT1 and C1GALT1C1 were assessed. Consistent with our observed glycan changes, C1GALT1 showed higher mRNA expression levels in NH_4_Cl treated astrocytes for 72 h than the untreated astrocytes, whereas the mRNA expression of C1GALT1C1 was not affected (Fig. 4a). Inhibition of glutamine synthetase completely blocked the upregulation of C1GALT1 mRNA expression in NH_4_Cl treated astrocytes; However, inhibition of NADPH oxidase could not block the upregulation of C1GALT1 mRNA expression in all astrocytes treated with NH_4_Cl (Fig. 4b). The protein expression levels of C1GALT1 shown by western blotting were consistent with their mRNA expression (Fig. 4c). Altered expression of C1GALT1 would explain the changes in glycan structures associated with ammonia-induced, which is in a glutamine synthesis-dependent manner.

Ca^2+^ signaling is an important regulator of many functions of astrocytes [29]. We doubted whether Ca^2+^ signaling might be subjected to ammonia regulation, so we used Fluo-4 AM to detect the Ca^2+^ in SVG p12 and SW1088 cells. The results demonstrated that after NH_4_Cl treatment, the intensity of Ca^2+^ was significantly stronger (Fig. 4d). Ca^2+^ homeostasis in astrocytes is regulated by key components of Ca^2+^, including mGluR5 and IP3R1 [16]. To determine whether the up-regulated Ca^2+^ was related to the expression of mGluR5 and IP3R1, their expression levels in astrocytes were examined by real-time PCR and western blotting. The mRNA expression of IP3R1 and mGluR5 was up-regulated in SVG p12 and SW1088 cells after NH_4_Cl was treated for 72 h (Fig. 4e). Only the protein expression level of IP3R1 was up-regulated in SVG p12 and SW1088 cells after NH_4_Cl treatment, while the protein expression level of mGluR5 was not up-regulated (Fig. 4e). These data strongly suggest that upregulation of ammonia regulated calcium homeostasis in astrocytes according to the expression of IP3R1.

### C1GALT1 silencing decreased ammonia-induced calcium activity in astrocytes

To determine whether ammonia-induced C1GALT1 is involved in regulating Ca^2+^ activity, we silenced C1GALT1 in the SVG p12 and SW1088 cells. In brief, cell lines were stably transduced with two independents short siRNAs targeting C1GALT1 (siC1GALT1-1 or siC1GALT1-2) or a non-targeting control (siNTC). The transfection efficiency was determined by negative control FAM (Additional file 1: Fig. S10). As expected, C1GALT1 knockdown, confirmed by both real-time PCR and western blotting (Fig. 4f). Then, we tested whether C1GALT1 was required for the expression of IP3R1. We found that both siC1GALT1-1 and siC1GALT1-2, but not siNTC, were able to restrain the expression of IP3R1 (Fig. 4g).

**Fig. 5:**
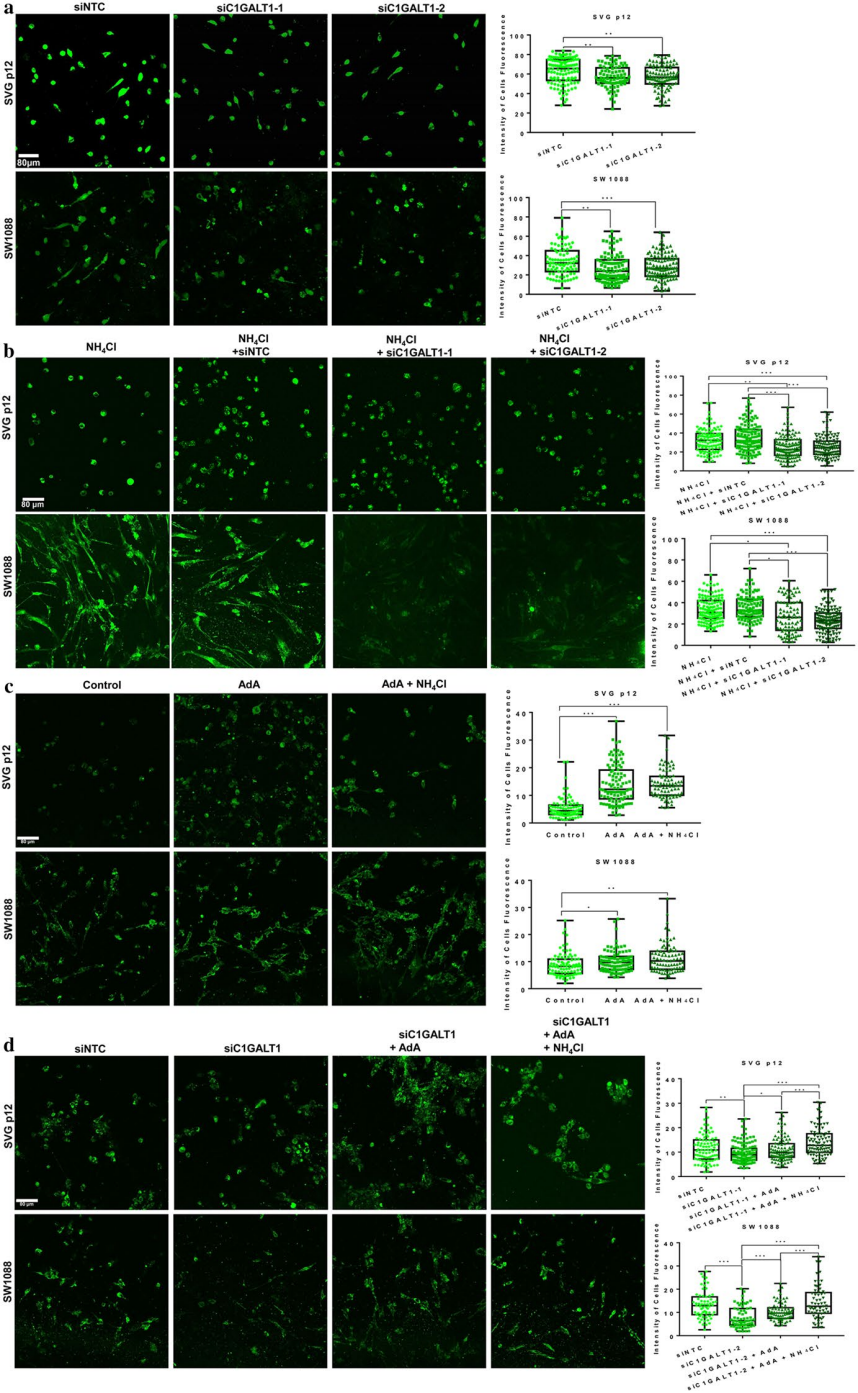
Ammonia regulated the expression of IP3R1 through C1GALT1 and affected calcium homeostasis. **a** Ca^2+^ was loaded with Fluo-4 AM in SVG p12 and SW1088 cells transduced with siC1GALT1-1, siC1GALT1-2 or siNTC and their fluorescence intensity of Ca^2+^. **b** Ca^2+^ in SVG p12 and SW1088 cells transduced with siC1GALT1-1, siC1GALT1-2 or siNTC plus NH_4_Cl and their fluorescence intensity of Ca^2+^. **c** Ca^2+^ in SVG p12 and SW1088 cells treated with AdA and NH_4_Cl, and their fluorescence intensity of Ca^2+^. **d** Ca^2+^ in SVG p12 and SW1088 cells transduced with siC1GALT1 plus AdA and NH_4_Cl, and their fluorescence intensity of Ca^2+^. Fluorescent images were captured using the same condition, Scale Bar 80 μm

The Ca^2+^ activity was significantly decreased by siC1GALT1-1 and siC1GALT1-2, but not siNTC, indicating that C1GALT1 may contribute to the Ca^2+^ activity in astrocyte (Fig. 5a). We next examined whether C1GALT1 knockdown could reverse ammonia-induced up-regulation of Ca^2+^ activity. We knocked down C1GALT1 and added NH_4_Cl in astrocytes simultaneously. The results showed that both siC1GALT1-1 and siC1GALT1-2, but not siNTC, could suppress ammonia-induced up-regulation of Ca^2+^ activity (Fig. 5b). Overall, ammonia-induced C1GALT1 regulated calcium activity.

### C1GALT1 decreased calcium activity by regulating IP3R1 expression in astrocytes

The previous results showed that C1GALT1 affected the expression of IP3R1. To determine whether IP3R1 was also involved in the regulation of Ca^2+^, we took the most potent known agonists of IP3R1 (Adenophostin A, AdA) to analyze the roles of IP3R1in Ca^2+^ dysbiosis induced by C1GALT1. This result suggested that AdA significantly increased intracellular Ca^2+^ in astrocytes, and AdA plus NH_4_Cl induced a slight increase in Ca^2+^ compared with AdA alone (Fig. 5c). In another similarly designed experiment, we found that AdA restored the down-regulation of Ca^2+^ induced by siC1GALT1, and AdA plus NH_4_Cl induced a more significant recovery in Ca^2+^ compared with AdA alone (Fig. 5d). In summary, C1GALT1 controlled Ca^2+^ homeostasis by regulating IP3R1.

### Calcium activity in the brain of HBV transgenic mice

**Fig. 6:**
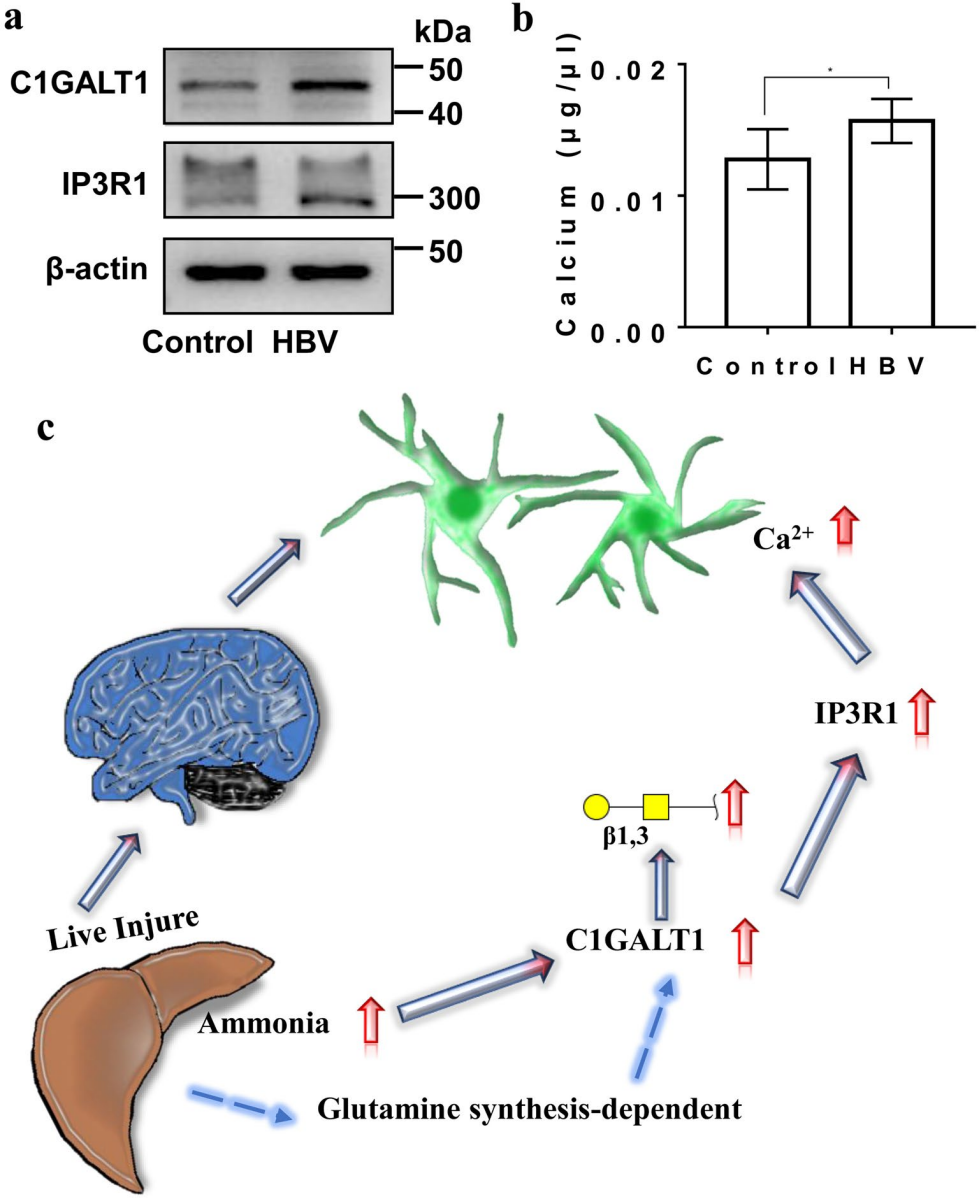
Calcium activity in the brain of HBV transgenic mice and a molecular network. **a** The protein expression levels of C1GALT1 and IP3R1 in the brains of 18 months old HBV transgenic mice and control mice. **b** Ca^2+^ concentration in the brains of 18 months old HBV transgenic mice and control mice. **c** A proposed molecular network, using lectin microarrays approach to assess glycosylation in brains of HBV transgenic mice and human astrocytes, find increased Galβ1-3GalNAc mediated by C1GALT1 in hyperammonemia and C1GALT1 facilitates calcium concentration due to the overexpression of IP3R1

Finally, in order to further explore whether C1GALT1, IP3R1 and related calcium expressions in the brain of HBV transgenic mice were consistent with the change trends in vitro experiments, we pooled brain tissue samples from six mice in each group to explore the calcium activity and the expression of related molecules in the brains of HBV transgenic mice. We noted that the protein expression levels of C1GALT1 and IP3R1 in the brains of 18 months old HBV transgenic mice were higher than those of control mice (Fig. 6a). In addition, Ca^2+^ concentration in the brains of 18 months old HBV transgenic mice were also higher than those of control mice (Fig. 6b). These results were consistent with what we observed in astrocytes.

## Discussion

Using HBV transgenic mice model, we demonstrated that blood ammonia concentration increased and also astrocytes swelled along with the process of liver injury. In the healthy liver, ammonia is metabolized by urea cycle. While the ammonia accumulated in the blood caused by advanced liver disease or portosystemic shunting can cross the blood–brain barrier and metabolize into glutamine in astrocytes; consequently, glutamine levels began to accumulate, leading to astrocyte swelling [30]. During the treatment of HE, reducing the production of ammonia and maximizing the body’s removal of ammonia are the main targets [31]. Astrocyte swelling is also a key feature of HE, and lowering ammonia levels can reduce brain swelling [3].

Although it has long been verified that protein glycosylation plays an important role in CNS, few glycomic analyses of HE. Here we have taken a systematic approach to identify protein glycosylation changes in the brain of HBV transgenic mice associated with chronic liver disease. Our analysis revealed the expression level of β-D-GlcNA, (GlcNAcβ1-4)_n_, Galβ1-4GlcNAc recognized by DSA, High-Mannose, Manα1-3Man, Manα1-6Man, Man5-GlcNAc2-Asn recognized by HHL, (GlcNAc)_2–4_ recognized by LEL, Fucα1-2Galβ1-4GlcNAc, Fucα1-3(Galβ1-4)GlcNAc, anti-H blood group specificity recognized by LTL, Fucα-1,6GlcNAc, α-D-Man, α-D-Glc recognized by LCA, Galβ1-3GalNAc recognized by MPL, High-Mannose, Manα1-6Man recognized by NPA, α-D-Man, Fucα-1,6GlcNAc, α-D-Glc recognized by PSA, Bisecting GlcNAc, biantennary complex-type N-glycan recognized by PHA-E, Terminal in GalNAc and Gal, anti-A and anti-B human blood group recognized by SJA, α-/β-linked terminal GalNAc, (GalNAc)_n_, GalNAcα1-3Gal, blood-group A recognized by SBA, and Terminating in GalNAcα/β1-3/6Gal reginized by WFA exhibited significantly changed in the brain protein of HBV transgenic mice compared with control mice.

The biosynthesis of glycans requires the synergism of glycosyltransferases and glycosidases [32]. There is currently little understanding of which enzymes are important in the biosynthesis underlying clinical pathologies, hindering the development of glycosylation-based therapeutic strategies [33]. Although ammonia is implicated with the urea cycle, astrocyte swelling, glutamine and gamma-amino-*n*-butyric acid systems in the pathogenesis of HE, the exact underlying mechanisms associated with glycosylation remain poorly understood [23]. We speculate that the abnormal glycosylation patterns observed in the brain of HBV transgenic mice could contribute to the ammonia concentration. Thus, we tested for impairment of ammonia on glycosylation by analyzing expression levels of protein glycosylation, and glycosyltransferases in NH_4_Cl treated astrocytes. Interestingly, except Galβ1-3GalNAc, the expression levels of other glycans did not alter consistently after ammonia stimulation. These data suggest that Galβ1-3GalNAc, mediated by glycosyltransferases of C1GALT1, has increased in ammonia-treated astrocytes within a glutamine synthesis-dependent manner. There is a single linear relationship between glycosyltransferase and glycan expression in some specific glycans coding pathways. However, due to the complementary coding of some glycosyltransferases, there is not a simple linear relationship between a single glycosyltransferase and its synthesis pathway, especially in the case of multiple factors, the regulation of glycosyltransferase is more complex.

Astrocytes release chemical messengers of Ca^2+^, suggesting that astrocytes play a more active role in brain function [34]. Moreover, astrocytes have been identified as the main targets of ammonium toxicity in the brain [35]. We next examined whether Ca^2+^ signaling might be subjected to ammonia regulation in astrocytes, and our results demonstrated that the intensity of Ca^2+^ was significantly stronger after ammonia treatment in astrocytes. Various plasma membrane receptors trigger Ca^2+^ signaling, and the key components of mGluR5 and IP3R1 are responsible for deregulates Ca^2+^ homeostasis in astrocytes [36]. Our data showed that upregulation of ammonia regulated Ca^2+^ homeostasis in astrocytes according to the overexpression of IP3R1. There is no established role for Galβ1-3GalNAc and neurotoxin ammonia in HE, and the importance of C1GALT1 to function of astrocytes is unknown. So, we next focused our functional studies on C1GALT1 as a candidate regulator of Ca^2+^ homeostasis in astrocytes. The results indicated that C1GALT1 silencing in astrocytes down-regulated the Ca^2+^ activity through the lower expression of IP3R1 in astrocytes, and C1GALT1 silencing could suppress ammonia induced up-regulation of Ca^2+^ activity.

## Conclusions

Together our data point toward that increased Galβ1-3GalNAc mediated by C1GALT1 in hyperammonemia and C1GALT1 facilitates Ca^2+^ concentration due to the overexpression of IP3R1, and a proposed molecular network is shown in Fig. 6c. A deep understanding of the underlying mechanism between glycosyltransferase and key factors of HE may offer more targeted therapeutic options in the future, and thus further research is necessary to fully understand the pathogenesis of HE.

## Materials and methods

### Animals

All mice experiments were performed in compliance with the People's Republic of China Legislation Regarding the Use and Care of Laboratory Animals and approved by the Laboratory Animal Ethics Committee of Northwest University and Fourth Military Medical University. HBV transgenic mice C57BL/6 J-TG(ALB1HBV)44BRI/J were provided by The Jackson Laboratory (Bar Harbor, ME) [17]. The negative control of C57BL/6 mice was purchased from VITALRIVER (Beijing, China). The HBV transgenic mouse and C57BL/6 mouse colonies were maintained in the Cell Engineering Research Centre and Department of Cell Biology of Fourth Military Medical University (Xi'an, China). HBV transgenic mice (n = 6, at the same age) and C57BL/6 mice (n = 6, at the same age) were anaesthetized and sacrificed at 4-, 6-, 8-, 10-, 12-, 14-, 16- and 18-months old for tissue material and serum material sampling.

### Cell lines and cell culture

Human brain astrocytes (SVG p12) and human astrocytoma cells (SW1088, CCF-STTG1) were verified by STR and maintained in mycoplasma-free conditions. SVG p12 cells were maintained in Dulbecco’s Modified Eagle’s Medium (DMEM, High Glucose; Hyclone, U.S.A) and CCF-STTG1 cells were maintained in RMPI 1640 medium (Hyclone, U.S.A) supplemented with 10% fetal bovine serum (FBS; Invitrogen, U.S.A) and 1% Penicillin–Streptomycin-Gentamicin Solution (Invitrogen, U.S.A) in a 37 ℃ incubator under 5% CO_2_. SW1088 cells were maintained in Leibovitz L-15 containing 10% FBS and 1% Penicillin–Streptomycin-Gentamicin Solution (Invitrogen, U.S.A) in a 37 ℃ incubator under humidified atmosphere.

### FFPE primary and H&E staining

The formalin-fixed paraffin-embedded (FFPE) tissues were prepared as previously described [37]. Briefly, fresh olfactory bulb, left hemisphere, right hemisphere, midbrain, cerebellum and lung tissues of mice were fixed in 4% paraformaldehyde. Then, they were dehydrated in ethanol and transparentized in a mixture of xylene with ethanol (1:1) followed by xylene. Finally, they were embedded in paraffin wax. All of the FFPE tissues were cut into 4 μm thick and attached to the glass slides. Hematoxylin and eosin-stained slides (H&E) were assessed to detect tissue changes. Briefly, FFPE tissue sections were dewaxed and rehydrated successively by xylene, 100%, 95%, 90%, 80% and 70% ethanol. These dewaxed and rehydrated sections were stained with hematoxylin and eosin.

### ELISA

Serum ammonia concentrations in all mice at 4 time points were determined using ELISA kits (Jianglai, China), according to the manufacture’s protocols. Serums from mice at each time point were pooled respectively, and each sample was independently performed four times. The standard curve was obtained according to the standard substance, then the absorbance value of each sample was substituted to get the corresponding serum ammonia concentration.

### Tissue and cell protein extraction

The olfactory bulb, left hemisphere, right hemisphere, midbrain and cerebellum of each mouse were placed in glass tubes containing 1 mL of ice-cold tissue protein extraction reagent (Thermo, U.S.A). The tissue was fragmented by homogenate in a homogenate instrument (Ningbo Scientz Biotechnology, China) until solid materials were not visible. The proteins were obtained by collecting the supernatant after ultracentrifugation at 120,000×*g* for 15 min at 4 °C, then protease inhibitors (Complete Protease Inhibitor Cocktail; Roche, Switzerland) were added. Cells were lysed in cold RIPA buffer supplemented with protease inhibitors, and then the proteins were obtained by collecting the supernatant after ultracentrifugation at 120,000×*g* for 15 min at 4 °C.

### Lectin microarrays

The proteins were labeled with Cy3 fluorescent dye (GE Healthcare, U.S.A) and purified using Sephadex G-25 columns (GE Healthcare, U.S.A). Lectins were purchased from Sigma or Vector Laboratories. The lectin microarrays were produced as previously described [38]. Briefly, 37 lectins with different binding preferences to glycans were spotted on homemade epoxy silane-coated slides. After immobilization, the slides were blocked with a blocking buffer containing 2% BSA in 1 × PBS (0.01 mol/L phosphate buffer containing 0.15 mol/L NaCl, pH 7.4) for 1 h. Then, the blocked slides were incubated with Cy3-labeled proteins diluted in 120 μL of incubation buffer for 3 h at room temperature. After incubation, the microarrays were scanned using a Genepix 4000B confocal scanner (Axon Instruments, U.S.A) set at 70% photomultiplier tube and 100% laser power. The acquired images were analyzed at 532 nm for Cy3 detection by Genepix 3.0 software. The values of average background lower than the ± 1 SD were removed. The median of the effective data points for each lectin was globally normalized to the sum of medians of all effective data points for each lectin in a block. The normalized intensity (NFI) data of the parallel groups were compared with each other based on fold change, according to the following criteria: fold change > 1.5 or < 0.67 in pairs indicated up-regulation or down-regulation. *P*-values less than 0.05 were considered statistically significant.

### Fluorescence-based lectin histochemistry and cytochemistry

The lectins were labeled with Cy5 fluorescent dye (GE Healthcare, U.S.A) and purified using Sephadex G-25 columns (GE Healthcare, U.S.A). Dewaxed and rehydrated tissue sections were microwaved in 10 mM citrate buffer (pH 6.0) at 100 °C for 10 min and cooled at room temperature to eliminate endogenous peroxidase activity, and 5% bovine serum albumin and 0.08% Triton X-100 was used to block nonspecific staining at 25 °C for 1 h. The tissue sections were incubated with Cy5-labelled lectin at 4 °C overnight. Finally, sections were stained with 1 μg/ml of DAPI (Roche, Switzerland) for 10 min. Cells were pre-inoculated into the dish, and 4% paraformaldehyde was used to immobilize the cells for 10 min. Then 5% bovine serum albumin and 0.01% Triton X-100 were used to block nonspecific staining at 25 °C for 1 h. Cells were incubated with Cy5-labelled lectin at 4 °C overnight. Finally, sections were stained with 1 μg/ml of DAPI for 10 min. A laser scanning confocal microscope FV 1000 (Olympus, Japan) was used to obtain the images. The images were acquired using the same condition and shown on the same scale in the Cy5- and DAPI-merge channel. The average fluorescence intensities of the images were obtained by total fluorescence intensities of lectins binding with cells divided by the total area of cells.

### Protein microarrays

A protein microarray was produced by 72 individual tissue samples of olfactory bulb divided into 8-, 10-, 12-, 14-, 16- and 18-months old HBV transgenic mice and control mice. The tissue samples were dissolved to a concentration of 1 mg/ml. Each tissue sample was spotted twice on the homemade epoxy silane-coated slides with Stealth micro spotting pins (SMP-10B) by a Capital smart microarrayer. The layout of the protein microarray is shown in Additional file 1: Fig. S5a. After completing the protein microarray, the immobilized slide was rinsed twice with 1 × PBST and 1 × PBS for 5 min each. After cleaning, protein microarray was blocked with the blocking buffer (10 mM 1 × PBS, 500 mM Glycine, 2% BSA, 0.05% Tween-20) for 1 h at room temperature and then rinsed twice with 1 × PBST and 1 × PBS for 5 min each. Subsequently, the blocked microarray was incubated with 10 μg of Cy5-labelled LEL diluted in 0.6 mL of incubation buffer for 3 h at room temperature in the dark. After incubation, the slide was washed twice with 1 × PBST and twice with 1 × PBS for 5 min each. A Genepix 4000B confocal scanner took the fluorescence intensity under 70% photomultiplier tube and 100% laser power with a channel of 635 nm for Cy5 detection. The values less than average background ± 1 SD were removed from each data point.

### Lectin blotting

Equal amounts of protein were resolved by SDS-PAGE, transferred onto PVDF membranes, and blocked in Carbo-Free Blocking Solution (Vector, CA) for 1 h at room temperature. Cy5-labeled lectins were diluted in blocking buffer with gentle shaking overnight at 4 °C in the dark. The membranes were then washed twice each for 10 min with TBST and scanned by fluorescence channel (635 nm excitation/650LP emission) with the voltage of 800 PMT using a phosphorimager (Storm 840, Molecular, U.S.A).

### Western blotting

Equal amounts of protein were resolved by SDS-PAGE, transferred onto PVDF membranes, and blocked-in buffer (5% milk in TBST, 1 h, room temperature). Primary antibodies were diluted in blocking buffer, as follows: C1GALT1 (1:2000; Abcam, UK), IP3R1 (1:1000; Abclonal, China), mGluR5 (1:500; Abways, China), β-actin (1:1000; TDYbio, China). Secondary antibodies were mouse-HRP or rabbit-HRP (1:5,000; Bio-Rad, China). Blots were developed using Western ECL Blotting Substrate (BioRad, China) and imaged in Qinxiang imaging system (Qinxiang, China).

### RNA extraction and real-time PCR

Total RNA was extracted by Trizol from samples (Sigma, U.S.A) and quantified using Nanodrop 8000 (Implen, German). 1 μg of RNA was reverse transcribed to cDNA using PrimeScript™ RT Master Mix (TAKARA, Japan) with the manufacturer’s recommendations. Transcripts were quantified by real-time PCR using Power SYBR Green PCR MasterMix (Accurate Biology, China). Gene-specific primers were designed using PrimerBank (https://pga.mgh.harvard.edu/primerbank/). Gene-specific primers were purchased from Sangong Biotech, China (Additional file 1: Table S2). Cycle threshold values were normalized to those of the housekeeping genes GAPDH. The average for three independent cultures performed at least 3 times was plotted as relative transcript abundance.

### Transfection of siRNA

SiRNA primers were purchased from GenePharma, China (Additional file 1: Table S3). Transfection conditions were optimized by negative control FAM (GenePharma, China). GenMute™ (SignaGen Laboratories, U.S.A) transfection complexes with siRNA pools were generated manufacturer’s recommendations. Media was changed after 6–12 h incubation with complexes. Cells were used for RNA or protein extraction after initiation of transfection for 48/72 h.

### Ca^2+^ imaging

Cytosolic free Ca^2+^ concentrations were determined by imaging the fluorescent probe Fluo-4 AM (Beyotime, China). Cells were incubated with 5 nM Fluo-4 AM for 30–40 min at 37 °C. Then, cells were washed with PBS buffer and incubated at 37 °C for another 30 min to allow complete esterification of the probe. A laser scanning confocal microscope FV 1000 (Olympus, Japan/Leica, German) was used to obtain the images. The images were acquired using the same condition and shown on the same scale in the Fluo-4 AM channel. The calcium fluorescence intensity of each cell was corrected by the average background intensity and then divided by the cell area to obtain the fluorescence intensity of each cell.

### Rescue

Astrocytes were incubated with transfection complexes for 12 h, then changed fresh media containing 1% FBS and 5 mmol/L NH_4_Cl for 72 h. During this period, the media was changed daily to ensure the concentration of NH_4_Cl. AdA (SantaCruz, U.S.A) is the most potent known agonists of IP3R1. After loading with Fluo-4 AM, cells were stimulated with 3/6 μmol/L AdA. Then, Ca^2+^ was determined by a laser scanning confocal microscope.

### Calcium colorimetric assay

Ca^2+^ of the brain from mice was determined by a Calcium Ion Assay Kit (Beyotime, China). According to the manufacture’s protocols, the absorbance value was determined at 575 nm by colorimetric comparison with calcium standard samples.

## Supplementary Information

**Additional file 1: Table S1.** Sugar-binding specificities of the 10 altered lectins in the HBV transgenic mice. **Table S2.** Related to real-time PCR methods: primers sequence. **Table S3.** Related to transfection of siRNA methods: siRNA primers sequence. **Fig. S1.** H&E staining of mice. **a**–**b**, The H&E staining of olfactory bulb (**a**) and cerebellum (**b**) from HBV transgenic mice and control mice at 10 to 18 months old. Scale bar, 100 μm. **Fig. S2.** Layout of the lectin microarrays. The lectin microarrays included 37 lectin probes, and each lectin was spotted in triplicate per block, with quadruplicate blocks on one slide. Cy3-labeled BSA as a marker and BSA as negative controls. **Fig. S3.** Glycopatterns of olfactory bulb and cerebellum in HBV transgenic mice and control mice. **a**, The glycopatterns of Cy3-labeled olfactory bulb samples bound to the lectin microarrays and their NFIs. **b**, Heat map and hierarchical clustering of the 37 lectins in glycopatterns of olfactory bulb. **c**, Four lectins revealed significant differences glycopatterns in olfactory bulb between HBV transgenic mice and control mice. **d**, The glycopatterns of Cy3-labeled cerebellum samples bound to the lectin microarrays and their NFIs. **e**, Heat map and hierarchical clustering of the 37 lectins in glycopatterns of cerebellum. The NFIs for each lectin were summarized as the mean values ± SD. In the heat map and hierarchical clustering, the samples were listed in columns and the lectins were listed in rows, and the color of each square represented the expression levels relative to the other data (Red, high; green, low; black, medium). **Fig. S4.** Normalized fluorescent intensities (NFIs) for each lectin in mice. **a-c**, The NFIs of lectin microarrays in left hemisphere (**a**), right hemisphere (**b**) and midbrain (**c**) from HBV transgenic mice and control mice. The NFIs for each lectin are summarized as the mean values ± SD. **Fig. S5.** Protein microarrays and lectin blotting analyses. **a–b**, The results of protein microarrays from olfactory bulb of HBV transgenic mice and control mice (**a**) and their fluorescence intensity (**b**). **c**, The results of SDS-PAGE from olfactory bulb of HBV transgenic mice and control mice. **d-e**, LEL bond to protein of olfactory bulb from HBV transgenic mice and control mice (**d**) and their fluorescence intensity (**e**). Data were presented as mean ± SD. **Fig. S6.** The images of fluorescence-based lectin histochemistry. **a**, Cy-5 labeled BSA as negative controls showed no positive signal. Scar bar 80 μm. **b**–**c**, The High-Mannose, Manα1-6Man recognized by NPA exhibited strong binding to the nuclear and cytoplasmic regions of granulosa cells in olfactory bulb (**b**), while NPA mainly bond to the cytoplasmic regions in left hemisphere (**c**). **d**–**e**, The α-D-Man, Fucα-1,6GlcNAc, α-D-Glc recognized by PSA showed strong binding to the cytoplasmic and membrane areas in the olfactory bulb (**d**) and left hemisphere (**e**). **f**–**g**, Galactose type recognized by SBA showed strong binding to the cell membrane, and little binding to cytoplasmic regions in the left hemisphere (**f**) and midbrain (**g**). Scar bar, 40 μm. **Fig. S7.** The normalized fluorescent intensities (NFIs) for each lectin in astrocytes. **a**–**c**, The NFIs for 37 lectins in three astrocytes are summarized as the mean values ± SD in NH_4_Cl treated compared with untreated SVG p12 (**a**), SW1088 (**b**), and CCF-STTG1 (**c**) cells. The average background was subtracted, and values less than the average background as negative data. The median of the effective data for each lectin was globally normalized to the sum of medians of all effective data points for each lectin in a block. **Fig. S8.** The images of fluorescence-based lectin cytochemistry and lectin microarrays. **a,** Cy-5 labeled BSA as negative controls showed no positive signal. Scar bar 80 μm. **b**–**c**, The glycopatterns of a Cy3-labeled SVG p12 bound to the lectin microarrays **(b)** and their NFIs **(c)**. SVG p12 cells were either left untreated or exposed to NH_4_Cl for 72 h, followed by another incubation for 72 h in NH_4_Cl free culture medium. The NFIs of MPL are summarized as the mean values ± SD. The average background was subtracted, and values less than the average background as negative data. The median of the effective data for each lectin was globally normalized to the sum of medians of all effective data points for each lectin in a block. **Fig. S9.** The fluorescence-based lectin cytochemistry. **a**–**b**, The fluorescence-based lectin cytochemistry (**a**) and average fluorescence intensity (**b**) of Galβ1-3GalNAc binder MPL in CH_3_NH_3_Cl treated and untreated SVG p12 cells. The images were acquired using the same condition and shown on the same scale in the Cy5- and DAPI-merge channel. Data were presented as mean ± SD. Scar bar 80 μm. **Fig. S10.** The transfection efficiency in SVG p12 cells. The transfection efficiency of siRNA and transfection reagent was determined using negative control FAM. Scale Bar, 100 μm.

## Data Availability

The datasets generated during and/or analyzed during the current study are available from the corresponding author on reasonable request.

## Abbreviations

APO: Apocynin
AdA: Adenophostin A
C1GALT1: Core 1 β1,3-galactosyltransferase
Ca^2^^+^: Calcium
CNS: Central nervous system
HE: Hepatic encephalopathy
HBV: Hepatitis B virus
IP3R1: Inositol 1,4,5-trisphosphate receptor type 1
mGluR5: Metabotropic glutamate receptor 5
MSO: L-Methionine sulfoximine
NFIs: Normalized fluorescent intensities
NADPH: Nicotinamide adenine dinucleotide phosphate
