# Supplementary material for: Role of ammonia for brain abnormal protein glycosylation during the development of hepatitis B virus-related liver diseases
Source: Cell Biosci. 2022 Feb 14;12:16. doi: 10.1186/s13578-022-00751-4 (PMC8842931; doi:10.1186/s13578-022-00751-4)
Supplement: Supplementary file 1 — Additional file 1: Table S1. Sugar-binding specificities of the 10 altered lectins in the HBV transgenic mice. Table S2. Related to real-time PCR methods: primers sequence. Table S3. Related to transfection of siRNA methods: siRNA primers sequence. Fig. S1. H&E staining of mice. a–b, The H&E staining of olfactory bulb (a) and cerebellum (b) from HBV transgenic mice and control mice at 10 to 18 months old. Scale bar, 100 μm. Fig. S2. Layout of the lectin microarrays. The lectin microarrays included 37 lectin probes, and each lectin was spotted in triplicate per block, with quadruplicate blocks on one slide. Cy3-labeled BSA as a marker and BSA as negative controls. Fig. S3. Glycopatterns of olfactory bulb and cerebellum in HBV transgenic mice and control mice. a, The glycopatterns of Cy3-labeled olfactory bulb samples bound to the lectin microarrays and their NFIs. b, Heat map and hierarchical clustering of the 37 lectins in glycopatterns of olfactory bulb. c, Four lectins revealed significant differences glycopatterns in olfactory bulb between HBV transgenic mice and control mice. d, The glycopatterns of Cy3-labeled cerebellum samples bound to the lectin microarrays and their NFIs. e, Heat map and hierarchical clustering of the 37 lectins in glycopatterns of cerebellum. The NFIs for each lectin were summarized as the mean values ± SD. In the heat map and hierarchical clustering, the samples were listed in columns and the lectins were listed in rows, and the color of each square represented the expression levels relative to the other data (Red, high; green, low; black, medium). Fig. S4. Normalized fluorescent intensities (NFIs) for each lectin in mice. a-c, The NFIs of lectin microarrays in left hemisphere (a), right hemisphere (b) and midbrain (c) from HBV transgenic mice and control mice. The NFIs for each lectin are summarized as the mean values ± SD. Fig. S5. Protein microarrays and lectin blotting analyses. a–b, The results of protein microarrays from olfactory b [file 13578_2022_751_MOESM1_ESM.docx]

**Table S1** Sugar-binding specificities of the 10 altered lectins in the HBV transgenic mice

| **Lectin** | **Specificity** |
| --- | --- |
| DSA | β-D-GlcNAc, (GlcNAcβ1-4)_n_, Galβ1-4GlcNAc |
| HHL | High-Mannose, Manα1-3Man, Manα1-6Man, Man5-GlcNAc2-Asn |
| LTL | Fucα1-2Galβ1-4GlcNAc, Fucα1-3(Galβ1-4)GlcNAc, anti-H blood group specificity |
| MPL | Galβ1-3GalNAc |
| NPA | High-Mannose, Manα1-6Man |
| PSA | α-D-Man, Fucα-1,6GlcNAc, α-D-Glc |
| PHA-E | Bisecting GlcNAc, biantennary complex-type N-glycan |
| SJA | Terminal in GalNAc and Gal, anti-A and anti-B human blood group |
| SBA | α- / β-linked terminal GalNAc, (GalNAc)_n_, GalNAcα1-3Gal, blood-group A |
| WFA | Terminating in GalNAcα/β1-3/6Gal |

**Table S2** Related to real-time PCR methods: primers sequence.

| **Gene** | **Direction** | **Primer sequence (5' - 3')** |
| --- | --- | --- |
| GAPDH | Forward | CTGGGCTACACTGAGCACC |
|  | Reverse | AAGTGGTCGTTGAGGGCAATG |
| C1GALT1 | Forward | TCCTCTGTGGATCAGCAATAGG |
|  | Reverse | TTAGGCTGGGTGTCAACCTTT |
| C1GALT1C1 | Forward | GGCAATGACTTATCACCCCAA |
|  | Reverse | CCAAATGCCCTAAGGCGGTAT |
| mGluR5 | Forward | CTGGAAAGGATCAATTCAGACCC |
|  | Reverse | GCCCAATGACCCCTACTATGG |
| IP3R1 | Forward | ATTGCTGGGGACCGTAATCC |
|  | Reverse | TCCAATGTGACTCTCATGGCA |

**Table S3** Related to transfection of siRNA methods: siRNA primers sequence.

| **Gene** | **Direction** | **Chemical modification** | **Primer sequence (5' - 3')** |
| --- | --- | --- | --- |
| Negative control FAM | Sence | - | UGACCUCAACUACAUGGUUTT |
|  | Antisence | - | AACCAUGUAGUUGAGGUCATT |
| siNegative control  (siNTC) | Sence | - | UUCUCCGAACGUGUCACGUTT |
|  | Antisence | - | ACGUGACACGUUCGGAGAATT |
| siC1GALT1-1 | Sence | 2'Ome | CCAGCCUAAUGUUCUUCAUTT |
|  | Antisence |  | AUGAAGAACAUUAGGCUGGTT |
| siC1GALT1-2 | Sence | 2'Ome | GCCAACAUAAAGAUGAGAATT |
|  | Antisence |  | UUCUCAUCUUUAUGUUGGCTT |

**
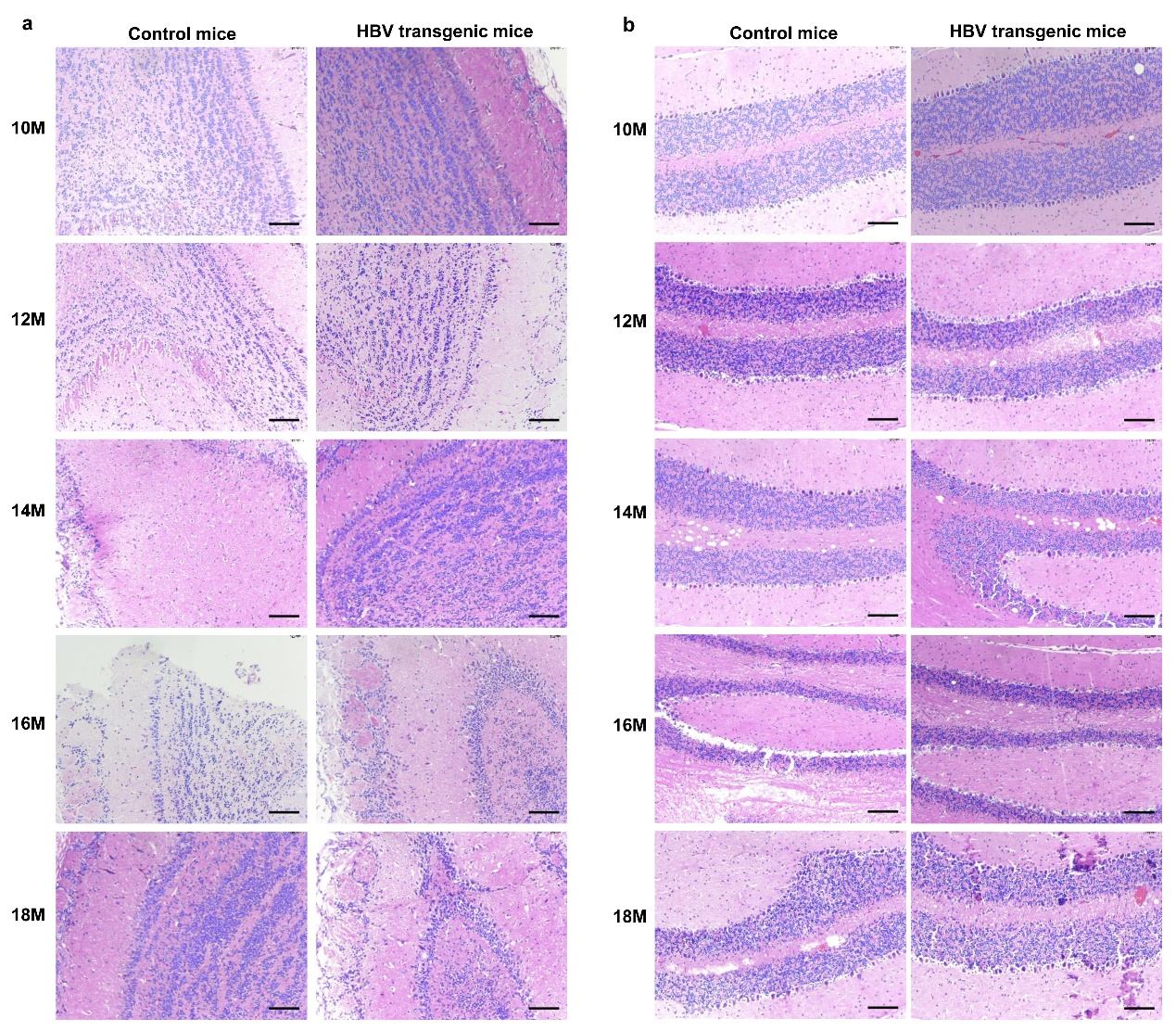
**

**Fig. S1** H&E staining of mice. **a-b**, The H&E staining of olfactory bulb **(a)** and cerebellum **(b)** from HBV transgenic mice and control mice at 10 to 18 months old. Scale bar, 100μm.


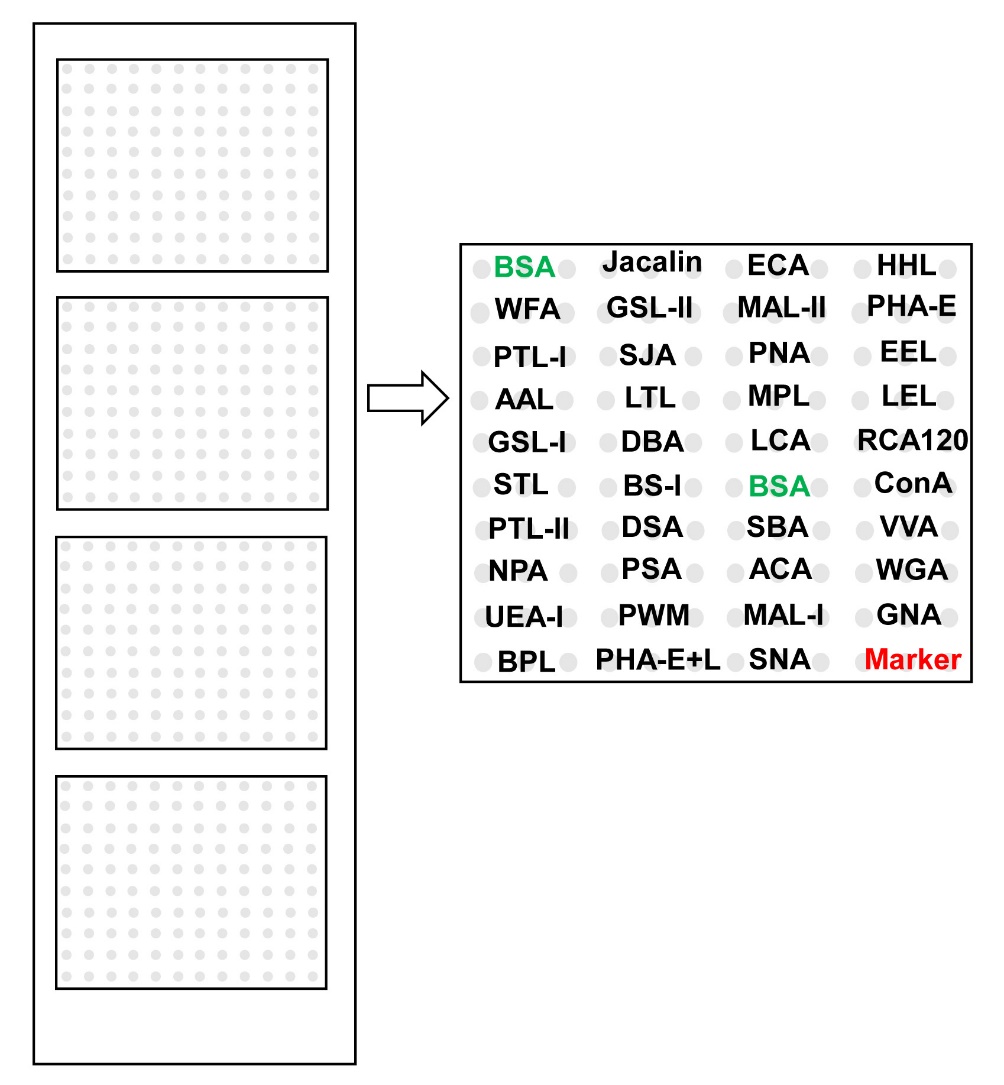


**Fig. S2** Layout of the lectin microarrays. The lectin microarrays included 37 lectin probes, and each lectin was spotted in triplicate per block, with quadruplicate blocks on one slide. Cy3-labeled BSA as a marker and BSA as negative controls.


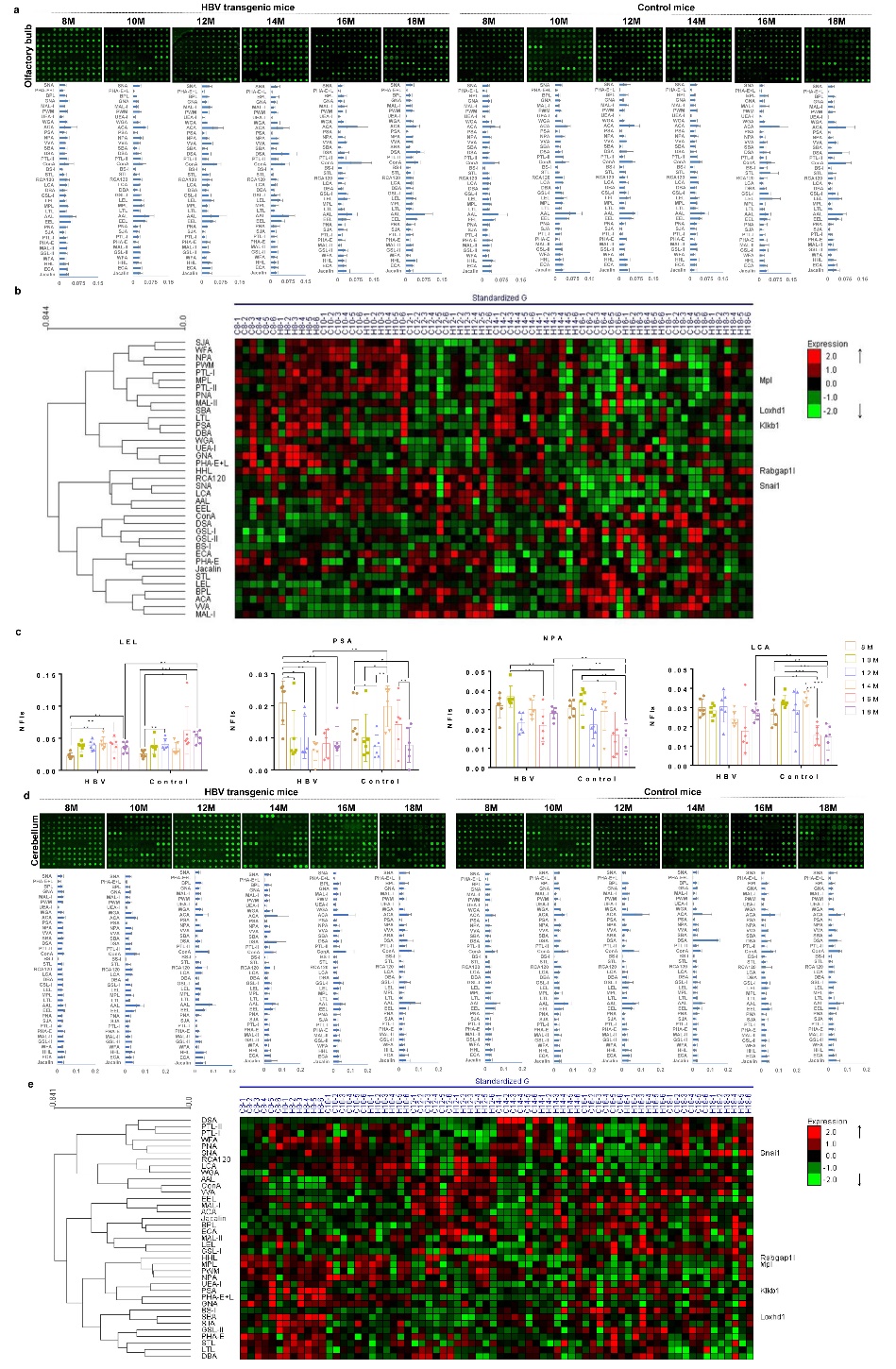


**Fig. S3** Glycopatterns of olfactory bulb and cerebellum in HBV transgenic mice and control mice. **a**, The glycopatterns of Cy3-labeled olfactory bulb samples bound to the lectin microarrays and their NFIs. **b**, Heat map and hierarchical clustering of the 37 lectins in glycopatterns of olfactory bulb. **c**, Four lectins revealed significant differences glycopatterns in olfactory bulb between HBV transgenic mice and control mice. **d**, The glycopatterns of a Cy3-labeled cerebellum samples bound to the lectin microarrays and their NFIs. **e**, Heat map and hierarchical clustering of the 37 lectins in glycopatterns of cerebellum. The NFIs for each lectin were summarized as the mean values ± SD. In the heat map and hierarchical clustering, the samples were listed in columns and the lectins were listed in rows, and the color of each square represented the expression levels relative to the other data (Red, high; green, low; black, medium).


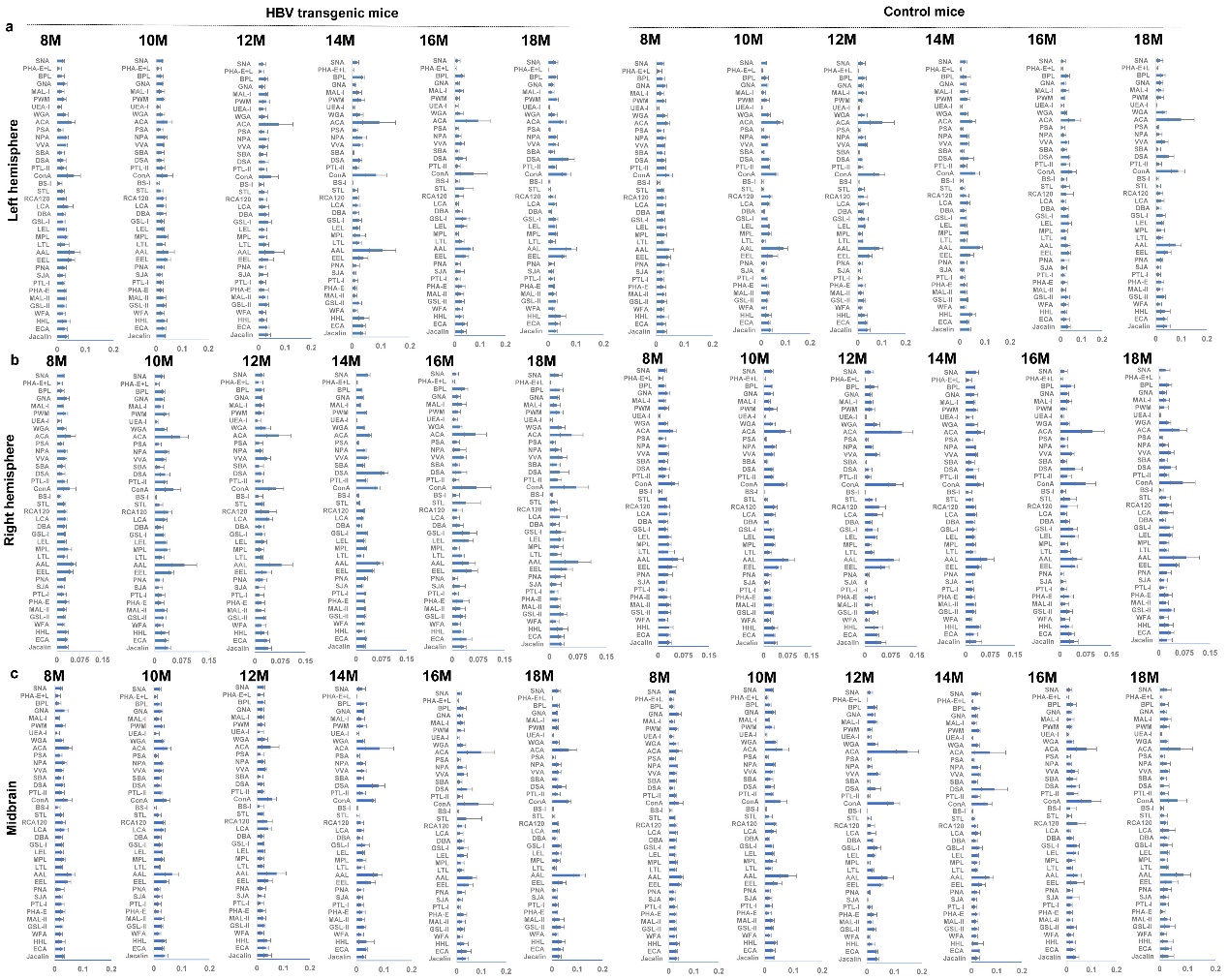


**Fig. S4** Normalized fluorescent intensities (NFIs) for each lectin in mice. **a-c**, The NFIs of lectin microarrays in left hemisphere (**a**), right hemisphere (**b**) and midbrain (**c**) from HBV transgenic mice and control mice. The NFIs for each lectin are summarized as the mean values ± SD.


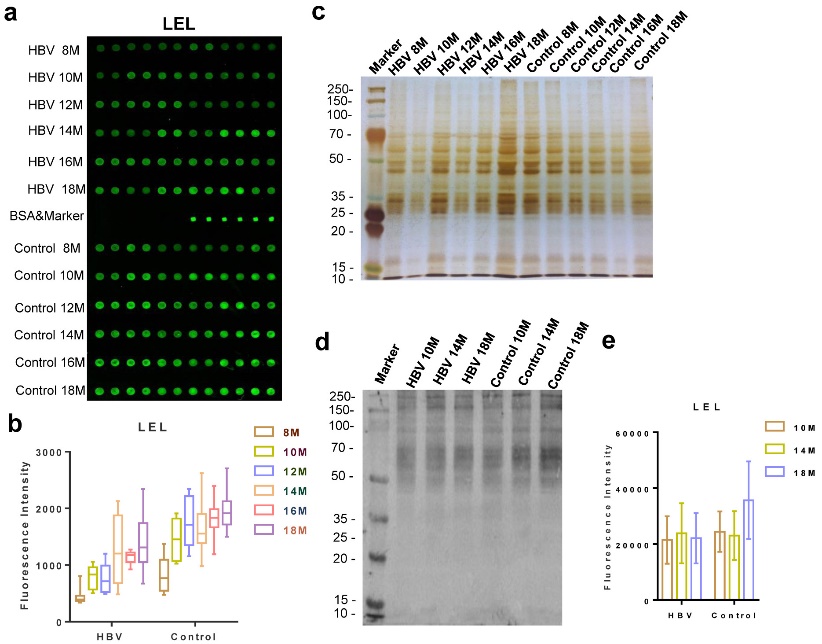


**Fig. S5** Protein microarrays and lectin blotting analyses. **a-b**, The results of protein microarrays from olfactory bulb of HBV transgenic mice and control mice (**a**) and their fluorescence intensity (**b**). **c**, The results of SDS-PAGE from olfactory bulb of HBV transgenic mice and control mice. **d-e**, LEL bond to protein of olfactory bulb from HBV transgenic mice and control mice (**d**) and their fluorescence intensity (**e**). Data were presented as mean ± SD.


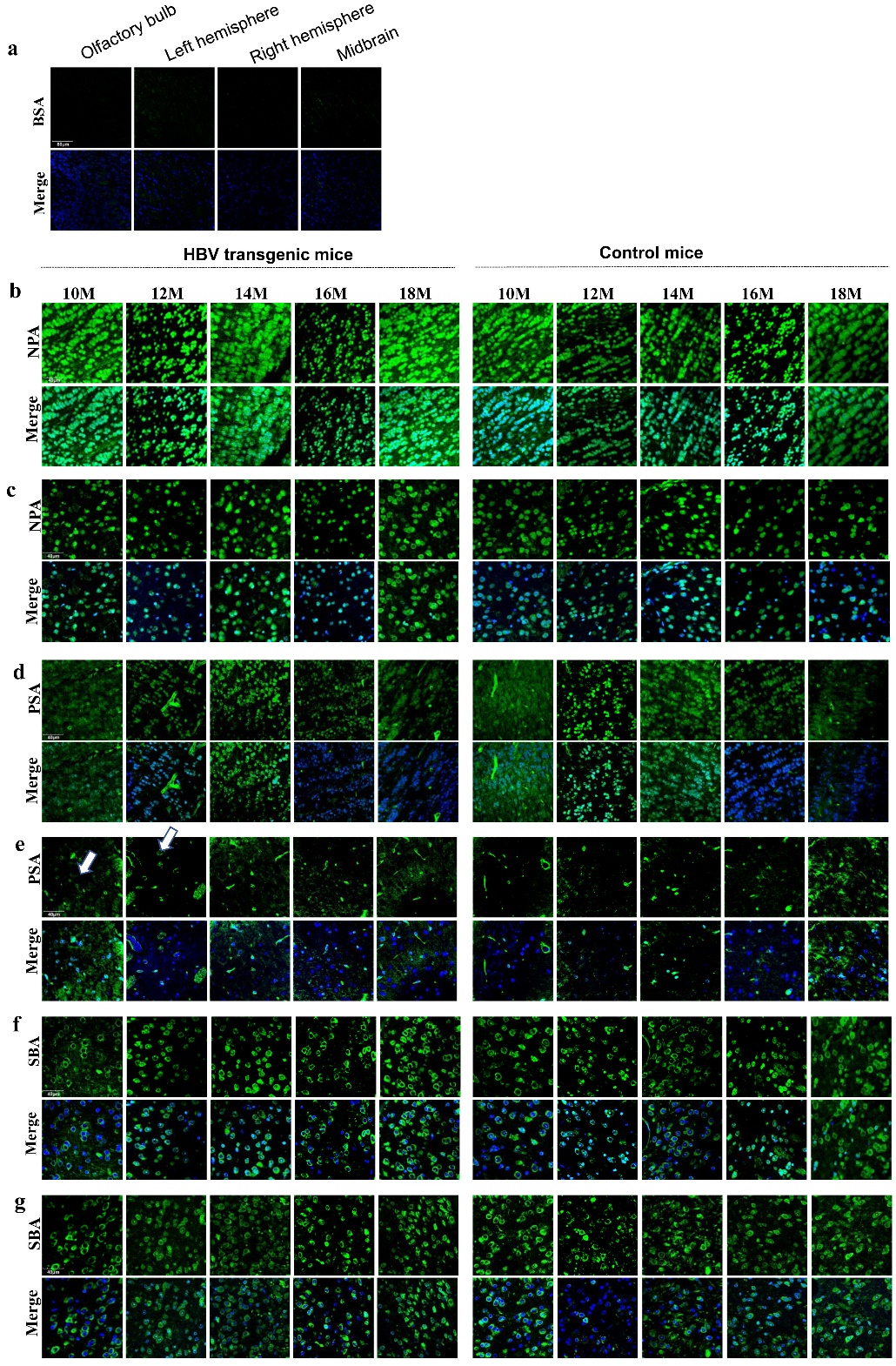


**Fig. S6** The images of fluorescence-based lectin histochemistry. **a**, Cy-5 labeled BSA as negative controls showed no positive signal. Scar bar 80μm. **b-c**, The High-Mannose, Manα1-6Man recognized by NPA exhibited strong binding to the nuclear and cytoplasmic regions of granulosa cells in olfactory bulb (**b**), while NPA mainly bond to the cytoplasmic regions in left hemisphere (**c**). **d-e**, The α-D-Man, Fucα-1,6GlcNAc, α-D-Glc recognized by PSA showed strong binding to the cytoplasmic and membrane areas in the olfactory bulb (**d**) and left hemisphere (**e**). **f-g**, Galactose type recognized by SBA showed strong binding to the cell membrane, and little binding to cytoplasmic regions in the left hemisphere (**f**) and midbrain (**g**). Scar bar, 40μm.


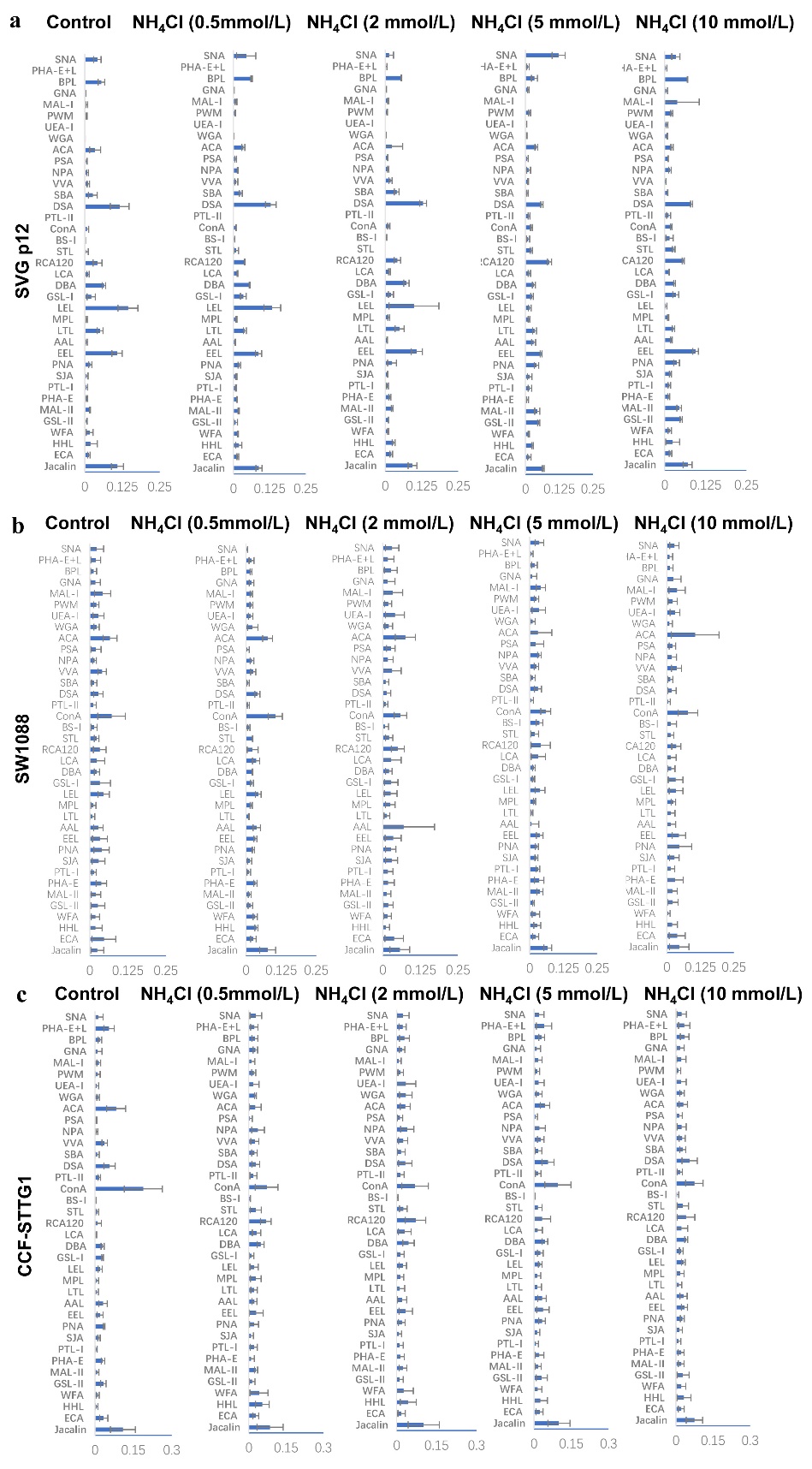


**Fig. S7** The normalized fluorescent intensities (NFIs) for each lectin in astrocytes. **a-c**, The NFIs for 37 lectins in three astrocytes are summarized as the mean values ± SD in NH_4_Cl treated compared with untreated SVG p12 (**a**), SW1088 (**b**), and CCF-STTG1 (**c**) cells. The average background was subtracted, and values less than the average background as negative data. The median of the effective data for each lectin was globally normalized to the sum of medians of all effective data points for each lectin in a block.


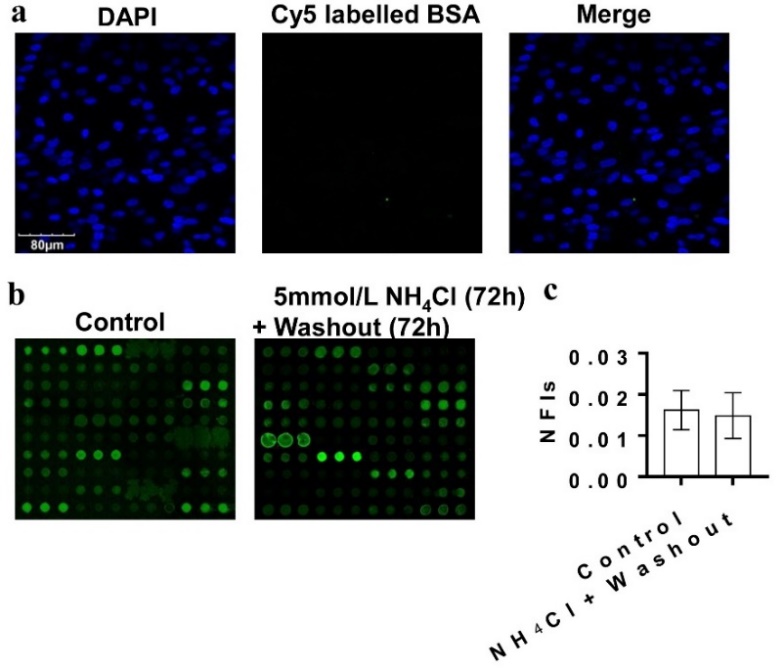


**Fig. S8** The images of fluorescence-based lectin cytochemistry and lectin microarrays. **a,** Cy-5 labeled BSA as negative controls showed no positive signal. Scar bar 80μm. **b-c**, The glycopatterns of a Cy3-labeled SVG p12 bound to the lectin microarrays **(b)** and their NFIs **(c)**. SVG p12 cells were either left untreated or exposed to NH_4_Cl for 72 h, followed by another incubation for 72 h in NH_4_Cl free culture medium. The NFIs of MPL are summarized as the mean values ± SD. The average background was subtracted, and values less than the average background as negative data. The median of the effective data for each lectin was globally normalized to the sum of medians of all effective data points for each lectin in a block.


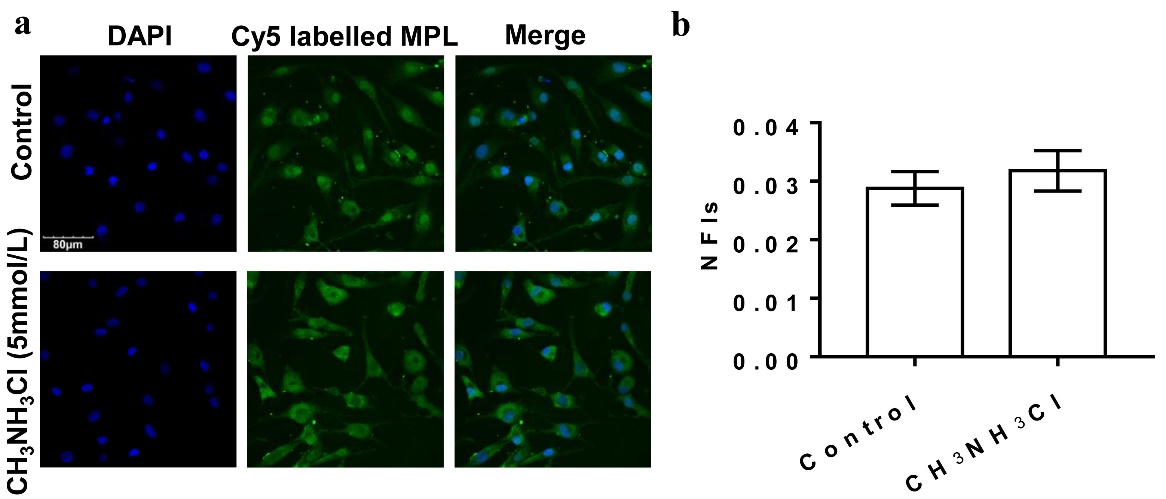


**Fig. S9** The fluorescence-based lectin cytochemistry. **a-b**, The fluorescence-based lectin cytochemistry (**a**) and average fluorescence intensity (**b**) of Galβ1-3GalNAc binder MPL in CH_3_NH_3_Cl treated and untreated SVG p12 cells. The images were acquired using the same condition and shown on the same scale in the Cy5- and DAPI-merge channel. Data were presented as mean ± SD. Scar bar 80μm


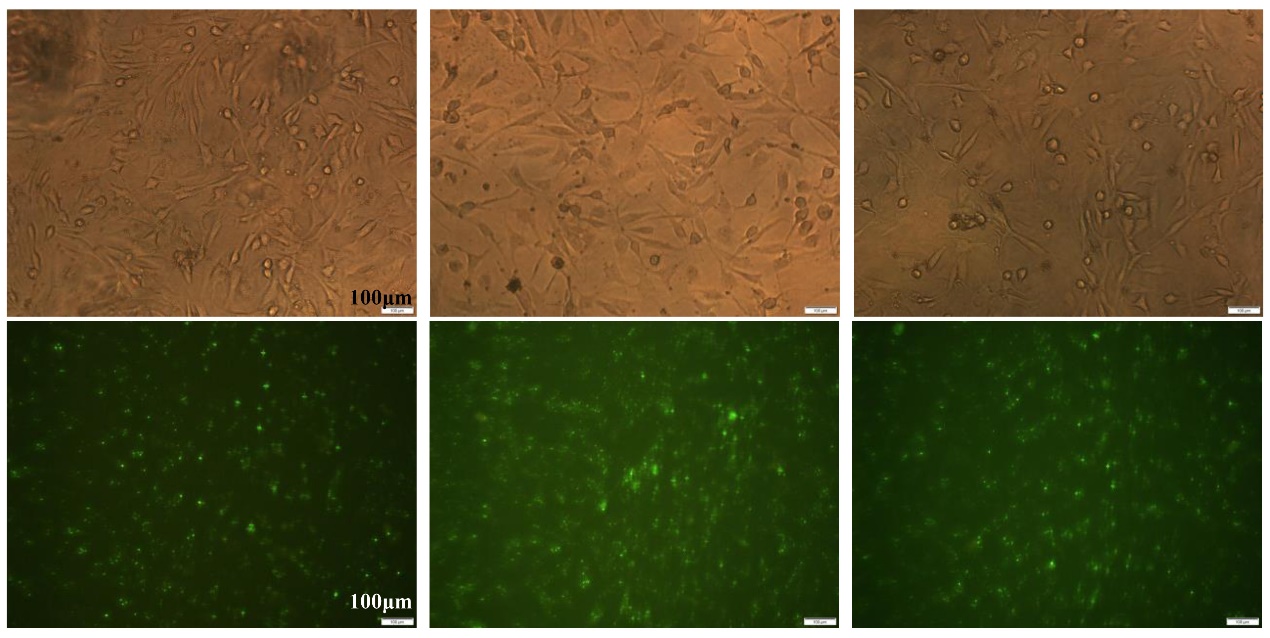


**Fig. S10** The transfection efficiency in SVG p12 cells. The transfection efficiency of siRNA and transfection reagent was determined using negative control FAM. Scale Bar, 100μm.
